# Chloroplast translational regulation uncovers nonessential photosynthesis genes as key players in plant cold acclimation

**DOI:** 10.1093/plcell/koac056

**Published:** 2022-02-16

**Authors:** Yang Gao, Wolfram Thiele, Omar Saleh, Federico Scossa, Fayezeh Arabi, Hongmou Zhang, Arun Sampathkumar, Kristina Kühn, Alisdair Fernie, Ralph Bock, Mark A Schöttler, Reimo Zoschke

**Affiliations:** Max Planck Institute of Molecular Plant Physiology, Potsdam-Golm, 14476, Germany; Max Planck Institute of Molecular Plant Physiology, Potsdam-Golm, 14476, Germany; Institut für Biologie, Martin-Luther-Universität Halle-Wittenberg, Halle (Saale), 06120, Germany; Max Planck Institute of Molecular Plant Physiology, Potsdam-Golm, 14476, Germany; Council for Agricultural Research and Economics, Research Center for Genomics and Bioinformatics (CREA-GB), Rome, 00178, Italy; Max Planck Institute of Molecular Plant Physiology, Potsdam-Golm, 14476, Germany; Institute of Optical Sensor Systems, German Aerospace Center (DLR), Berlin, 12489, Germany; Max Planck Institute of Molecular Plant Physiology, Potsdam-Golm, 14476, Germany; Institut für Biologie, Martin-Luther-Universität Halle-Wittenberg, Halle (Saale), 06120, Germany; Max Planck Institute of Molecular Plant Physiology, Potsdam-Golm, 14476, Germany; Max Planck Institute of Molecular Plant Physiology, Potsdam-Golm, 14476, Germany; Max Planck Institute of Molecular Plant Physiology, Potsdam-Golm, 14476, Germany; Max Planck Institute of Molecular Plant Physiology, Potsdam-Golm, 14476, Germany

## Abstract

Plants evolved efficient multifaceted acclimation strategies to cope with low temperatures. Chloroplasts respond to temperature stimuli and participate in temperature sensing and acclimation. However, very little is known about the involvement of chloroplast genes and their expression in plant chilling tolerance. Here we systematically investigated cold acclimation in tobacco seedlings over 2 days of exposure to low temperatures by examining responses in chloroplast genome copy number, transcript accumulation and translation, photosynthesis, cell physiology, and metabolism. Our time-resolved genome-wide investigation of chloroplast gene expression revealed substantial cold-induced translational regulation at both the initiation and elongation levels, in the virtual absence of changes at the transcript level. These cold-triggered dynamics in chloroplast translation are widely distinct from previously described high light-induced effects. Analysis of the gene set responding significantly to the cold stimulus suggested nonessential plastid-encoded subunits of photosynthetic protein complexes as novel players in plant cold acclimation. Functional characterization of one of these cold-responsive chloroplast genes by reverse genetics demonstrated that the encoded protein, the small cytochrome *b*_6_*f* complex subunit PetL, crucially contributes to photosynthetic cold acclimation. Together, our results uncover an important, previously underappreciated role of chloroplast translational regulation in plant cold acclimation.

## Teaser

Cold exposure induces translational regulation of plastid-encoded genes and reveals a critical role of a small nonessential cytochrome b6f complex subunit in chilling acclimation.

## Introduction

Plants have evolved complex mechanisms to acclimate to diverse environmental conditions, including low temperatures. A drop in temperature slows down all enzymatic reactions and affects phytohormone biosynthesis, thereby influencing plant growth, development, and eventually productivity. Chilling triggers multilayered cellular acclimation processes such as modifications of membrane structure and composition, changes in calcium signaling and osmolyte accumulation, reprogramming of gene expression, and metabolism as well as modifications in photosynthesis (Theocharis et al., 2012).

IN A NUTSHELL
**Background:** Plants have to cope with ever-changing environmental conditions. Climate change causes sudden cold periods more frequently, especially in the spring. Rapidly dropping temperatures trigger several acclimation responses in plants, including changes in gene expression. Plants contain genomes within the nucleus, but also in mitochondria and chloroplasts (organelles of endosymbiotic origin). While cold-induced alterations in nuclear gene expression have been studied in detail and functionally linked to acclimation responses, close to nothing is known about the contribution of chloroplast genes to cold acclimation.

**Questions:** Employing genome-wide approaches, we asked how chloroplast transcription and translation are altered during chilling and which chloroplast genes are involved in plant cold acclimation.

**Findings:** Within 2 days of chilling, chloroplast gene expression in the model plant tobacco is mainly altered at the level of translation, while transcription does not substantially respond to cold. We identified 13 chloroplast genes whose translation is either induced or repressed by cold and mainly encode nonessential subunits of the photosynthesis machinery. These nonessential genes are dispensable for growth at ambient temperature. For one such gene, *petL*, encoding a small, nonessential subunit of the cytochrome *b*_6_*f* complex (involved in the electron transport reactions of photosynthesis), we show that its expression is crucially required for adequate cold acclimation of the light reactions of photosynthesis. Consequently, plants lacking petL display impaired photosynthesis and bleached leaves in the cold.

**Next steps:** We want to understand how *petL* and other cold-responsive chloroplast genes are regulated during cold acclimation: which factors control their cold-regulated translation and which RNA elements are involved in the molecular mechanisms of chloroplast cold regulation? Furthermore, we want to decipher how cold-responsive chloroplast genes do strengthen photosynthesis, chloroplasts and the whole plant during chilling. This knowledge will support efforts to create new crop plant varieties that can withstand adverse temperature conditions, including unexpected cold periods.

Chloroplasts have been suggested to act as central sensors of and responders to low temperatures because the above-mentioned effects originate from chloroplasts, involve them and/or directly affect them (Svensson et al., 2006; Crosatti et al., 2013; Kleine et al., 2021). In the short term, low temperatures generate imbalances in photosynthesis by decelerating enzymatic reactions of the Calvin–Benson–Bassham (CBB) cycle while virtually not directly affecting electron transport of the light reactions. Light harvesting and energy transfer to the reaction centers of photosystems II and I (PSII and PSI) are fully active at low temperatures (Crosatti et al., 2013), while cold slows the metabolic consumption of NADPH and ATP by the CBB cycle and other energy-consuming processes (Huner et al., 1998). Thus, chilling disrupts redox and energy homeostasis and causes higher PSII excitation pressure. In this situation, PSII is quasi-immediately protected by reducing energy transfer to the reaction center by nonphotochemical quenching. Within minutes to days, PSII repair is stimulated and antenna size decreases, the stoichiometry of photosynthetic complexes is adjusted and photochemical quenching restored (Huner et al., 1998; Schöttler et al., 2017). Together, these changes minimize the deleterious effects of photoinhibition and overproduction of reactive oxygen species (Mittler, 2002). Furthermore, the cold-induced drop of other metabolic reactions such as sucrose biosynthesis inhibits photosynthesis (Hurry et al., 2000). Chilling triggers additional acclimatory adjustments in primary metabolism, including accumulation of organic acids, amino acids and sugars (Fürtauer et al., 2019). Moreover, within hours of cold exposure, thylakoid membranes swell and distort, starch granules decrease in number and size (Kratsch and Wise, 2000), and chloroplasts move to avoid excessive light (Fujii et al., 2017).

To orchestrate cold acclimation processes, nuclear gene expression responds to low temperatures (Garcia-Molina et al., 2020). The C-repeat binding factor cold response pathway constitutes a regulatory hub (Thomashow, 2010) that is activated by cryo-sensing cytosolic 80S ribosomes (Guillaume-Schöpfer et al., 2020) and coordinately induces cold-responsive genes (Jaglo-Ottosen et al., 1998; Zhao et al., 2016), including many that encode chloroplast-targeted proteins (Crosatti et al., 2013; Garcia-Molina et al., 2020). Photosynthetic core subunits, however, are plastid-encoded and long-term chilling causes lower chloroplast transcript levels, overaccumulation of unprocessed transcript precursors and altered translation elongation (Kupsch et al., 2012; Grennan and Ort, 2007). Additionally, conditional cold-sensitive phenotypes of mild plastid translation mutants suggest that a fully functional chloroplast translation system is crucial for chilling acclimation (e.g. Barkan, 1993; Rogalski et al., 2008; Liu et al., 2010; Fleischmann et al., 2011; Wang et al., 2016). However, although translational adjustments dominate the regulation of plastid gene expression (Zoschke and Bock, 2018), a comprehensive, time-resolved analysis of cold-induced responses has been missing for chloroplast gene expression, such that roles of chloroplast translational regulation in plant chilling acclimation have remained unknown.

We report here a systematic genome-wide analysis of chloroplast gene expression in tobacco (*Nicotiana tabacum*) over 2 days of cold acclimation. Our data revealed fast and extensive adjustments of chloroplast translation for specific plastid genes at the initiation and elongation stages. At the same time, transcript levels remained virtually unchanged. Interestingly, most cold-responsive genes are not essential for autotrophic growth at ambient temperature. We demonstrate that PetL, a translationally cold-induced nonessential cytochrome *b*_6_*f* (cyt *b*_6_*f*) subunit, overaccumulates in comparison to the core cyt *b*_6_*f* complex during cold acclimation. Furthermore, our results revealed that PetL is crucial for effective photosynthetic performance under prolonged exposure to low temperature. In summary, our data (1) reveal that dynamic translational reprogramming predominates the adjustment of chloroplast gene expression to low temperature and (2) uncover an important role of chloroplast translational regulation in plant chilling acclimation.

## Results

### Low temperature provokes photosynthetic, cellular, and metabolic acclimation responses in tobacco

**Figure 1:**
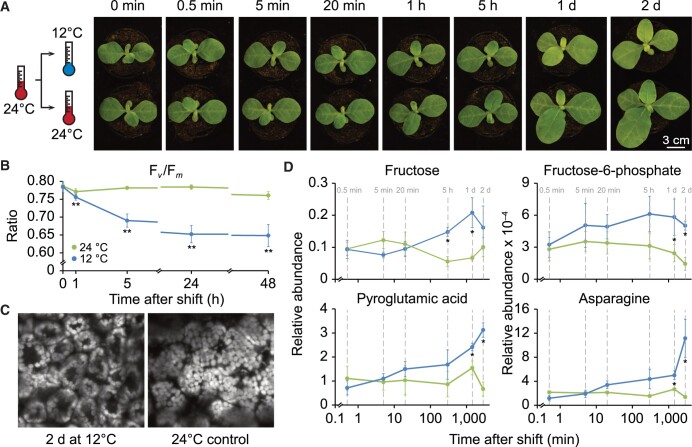
Design of the temperature-shift experiment and observed phenotypic, photosynthetic, cellular, and metabolic alterations during cold acclimation of tobacco seedlings. A, Left: schematic diagram representing the experimental design of the cold-shift experiment. Right: visible phenotypes of acclimating and control plants at the indicated time points after shift (shift was performed 21 days after sowing). B, Photosynthetic performance during cold acclimation, as indicated by maximum quantum efficiency of PSII in the dark-adapted state (F_*v*_/F_*m*_) for acclimating and control plants. Two different leaf areas of four acclimating and four control plants were measured (*n* = 8). Asterisks indicate statistically significant differences between acclimating and control seedlings (**P <0.01, two-sided Student’s *t* test, Supplemental Data Set 1). C, Cold-induced chloroplast movements. A top–down view of chloroplasts in mesophyll cells of acclimating and control plants grown for 2 days after cold shift. Images are representative overlays comprising several optical sections imaged by confocal microscopy. Three additional independent biological replicates (from individual plants) are shown in Supplemental Figure S2. Note the cold-induced movement of chloroplasts from the cells periclinal to anticlinal face. D, Cold-induced alterations of specific primary metabolites at the indicated time points (marked by gray vertical dashed lines). Error bars denote the standard deviation of results from at least three individual plants used as independent biological replicates. Asterisks indicate statistical significance (*q*-value < 0.1, Supplemental Data Set 2) >1.5-fold changes between acclimating and control plants. Primary metabolites, whose relative abundances changed >1.5-fold with statistical significance for at least two consecutive time points, are shown here, and cold-induced changes for additional primary and secondary metabolites are shown in Supplemental Figure S3.

In plant physiology, low temperatures are defined as any nonoptimal above-freezing temperatures (Theocharis et al., 2012) that trigger numerous processes referred to as cold or chilling acclimation (Kleine et al., 2021). To examine these responses in the dicotyledonous model plant tobacco, we shifted 3-week-old wild-type (WT) seedlings from moderate to low growth temperature (24°C to 12°C; Figure 1A). At 12°C, tobacco can efficiently cold-acclimate, as demonstrated by its ability to largely maintain growth (Figure 1A). At the selected young developmental stage of 3 weeks, the biogenesis of photosynthetic complexes is still ongoing, thereby enabling high flexibility in response to changing environmental conditions, especially at the level of gene expression. After the downshift of environmental temperature, leaf temperature decreased rapidly with most of the temperature change transmitted to leaves within 2 min (Supplemental Figure S1). To minimize potential circadian or developmental effects on the results, we maintained a control group of plants that was constantly grown at moderate growth temperature (Figure 1A). Prolonged exposure to low temperature (≥1 days) induced visible phenotypic changes, namely a slightly pale green phenotype and mild growth retardation (Figure 1A). Consequently, to ensure exactly matching developmental stages between acclimating and control plants, we restricted the subsequent comparative analysis of chloroplast gene expression to a maximum of 2 days after the shift.

We observed a significant (*P* < 0.01) decrease in maximum quantum efficiency of PSII in the dark-adapted state (F_*v*_/F_*m*_) starting at 1 h in low temperature and reached a minimum after 2 days in the cold (0.65 and 0.76 in cold acclimating and control plants, respectively; Figure 1B). Lower F_*v*_/F_*m*_ indicates mild PSII photoinhibition, a well-described effect of low temperature (Harvaux and Kloppstech, 2001). Longer exposure to cold (>1 days) did not cause any further significant reduction of F_*v*_/F_*m*_. Microscopic analysis revealed that low temperature triggers a light avoidance-like movement of chloroplasts from the periclinal to the anticlinal cell face (Figure 1C; Supplemental Figure S2), a response that is known to support the protection of photosynthetic light reactions from excessive light during cold acclimation (Fujii et al., 2017).

To examine the dynamics of primary and secondary metabolites in cold-acclimating leaves, we applied gas chromatography and high-resolution liquid chromatography mass spectrometry. These analyses revealed numerous adjustments in metabolite levels during cold acclimation (Figure 1D;  Supplemental Figure S3). We detected significant cold-triggered increases (>1.5-fold), for instance in the levels of fructose and fructose-6-phosphate (Figure 1D). Fructose-6-phosphate levels constantly increased in cold-acclimating plants (with a maximum 3.5-fold increase after 2 days at low temperature). Elevated contents for fructose sugars are a known metabolic cold acclimation strategy that is embedded in a wider antioxidative cold response (Bogdanović et al., 2008). Furthermore, after prolonged exposure to low temperature, we detected significantly (*q*-value < 0.1) increased levels for the amino acids alanine, asparagine, pyroglutamic acid, glutamate, lysine, ornithine, and the amino acid derivative putrescine (Figure 1D;  Supplemental Figure S3). Elevated accumulation of amino acids has been associated with various mechanisms of metabolic cold acclimation, especially in anticipation of subsequent cold stress (Obata and Fernie, 2012). Higher levels of pyroglutamic acid (5-oxo-L-proline), a glutathione derivative, for example, are related to protein degradation that delivers small osmoregulatory molecules (Cook et al., 2004; Guy et al., 2008; Xu et al., 2020).

Together, these results validate the applied temperature shift as inducing a general physiological cold acclimation response in tobacco seedlings, which is below the threshold of permanently damaging cold stress.

### Mildly reduced global ribosome coverage during cold acclimation

**Figure 2:**
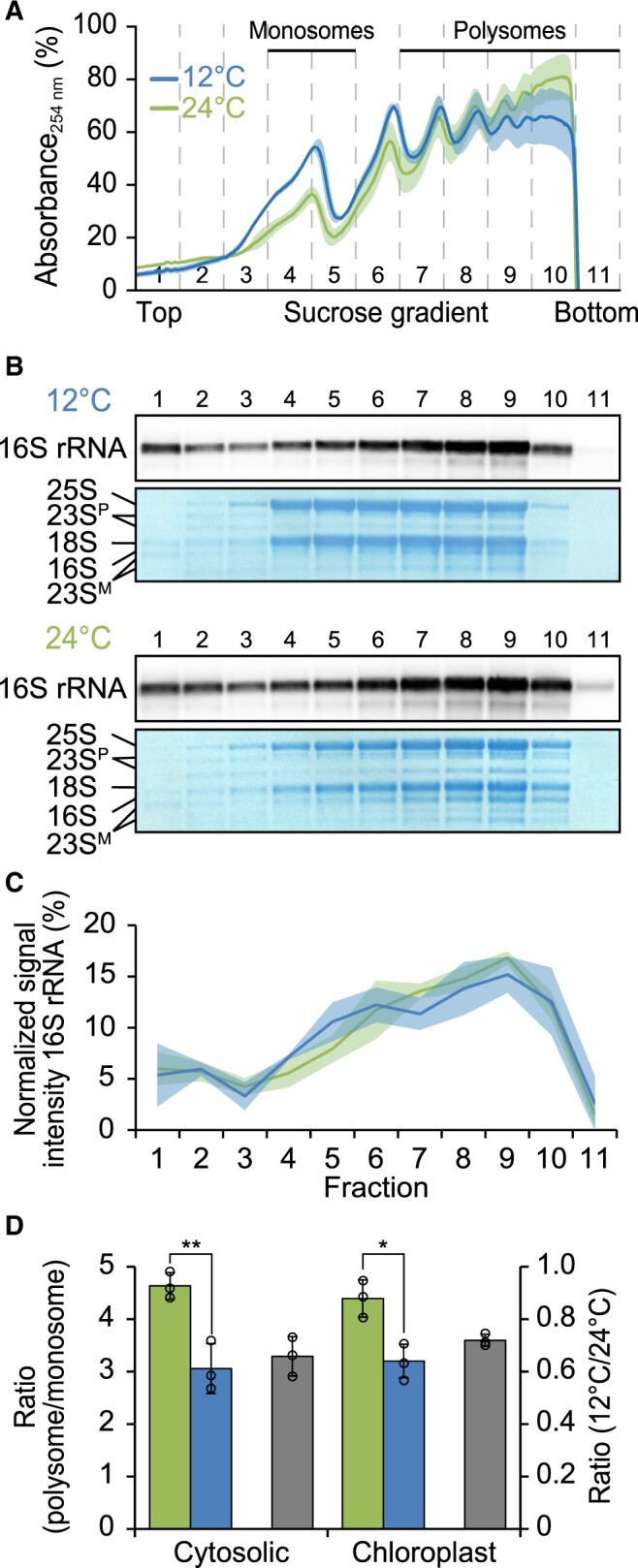
Mild global reduction of cytosolic and chloroplast translation initiation after 2 days at low temperature. A, Polysome profiles of cytosolic ribosomes in acclimating (12°C, blue) and control (24°C, green) plants (this color code is used throughout the figure). Absorbance at 254 nm is given in percent of the maximum absorbance, shading represents the standard deviation of results obtained from three individual plants used as independent biological replicates (note that cytosolic ribosomes predominate the profiles while chloroplast ribosomes represent a relatively minor fraction). Sucrose density gradients were separated into 11 fractions of equal volume as indicated by vertical dashed lines. The fractions containing monosomes and polysomes are labeled. B, Representative RNA gel blot analyses demonstrating the distribution of the chloroplast 16S rRNA in fractions recovered from the gradients shown in (A) for both acclimating and control plants. Numbers above the blots denote the gradient fractions. Methylene blue staining of rRNAs is shown as control for equal loading. 23S^P^, chloroplast 23S rRNA precursors; 23S^M^, generated by hidden-break processing). C, Quantification of 16S rRNA levels in each fraction for acclimating and control plants, normalized to the sum of all signals across fractions, plotted as a function of gradient fraction (to visualize chloroplast ribosome distributions). Shading represents the standard deviation based on three individual plants used as independent biological replicates. D, Bar plots showing the ratio between polysomal and monosomal rRNAs as a measure of global cytosolic and chloroplast translation initiation activity at 24°C and 12°C (left ordinate, green and blue bars, respectively) as well as the relative translation initiation activity in cold compared to control conditions (right ordinate, gray bars). Error bars denote the standard deviation of results obtained from three individual plants used as independent biological replicates. Asterisks indicate statistically significant differences between acclimating and control plants (*P < 0.05, **P < 0.01, two-sided Student’s *t* test, Supplemental Data Set 1).

**Figure 3:**
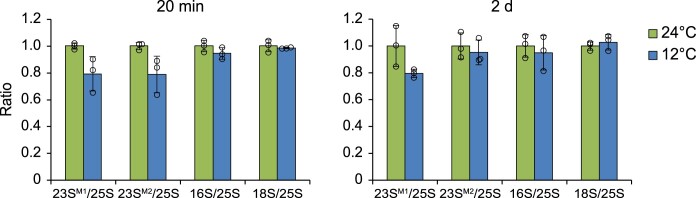
Relative accumulation of cytosolic and chloroplast rRNAs as a measure for the accumulation of the corresponding ribosomal subunits during cold acclimation. Bar plots showing the relative accumulation of rRNAs (normalized to 25S rRNA) from acclimating plants (12°C, blue) and control plants (24°C, green) at the indicated time points after cold shift. The accumulation of nuclear 25S rRNA and 18S rRNA, chloroplast 23S rRNAs (23S^M1^:∼1 kb; 23S^M2^:∼1.2 kb, representing two of the three hidden break products (Kössel et al., 1985)), and 16S rRNA were quantified with 2100 Agilent Bioanalyzer and displayed as rRNA ratios by normalizing to 25S rRNA. Error bars denote the standard deviation of results obtained from three individual plants used as independent biological replicates. Note that no statistically significant differences were observed between acclimating and control plants (Supplemental Data Set 1).

Based on the van’t Hoff rule (the rate of a reaction decreases two-fold for any 10°C-drop in temperature) and examinations in plants (Guillaume-Schöpfer et al., 2020) and bacteria (Farewell and Neidhardt, 1998), a temperature reduction by 12°C decelerates translation elongation and therefore protein synthesis rates globally two- to three-fold. This effect should be nondiscriminative and should affect translation elongation rates equally on all transcripts. In bacteria, cold shock additionally induces a widespread block of translation initiation (Weber and Marahiel, 2003). To test for similar effects in plants, we comparatively analyzed overall ribosome loading by polysome analysis for an early (20 min) and a late (2 days) time point during cold acclimation (Figure 2;  Supplemental Figure S4). Considering translation initiation as the rate-limiting step for ribosome loading (Shah et al., 2013), polysome analysis provides a qualitative measure of translation initiation by determining ribosome coverage of mRNAs through migration behavior in sucrose gradients. UV distribution measurements of the dominating rRNAs of cytosolic 80S ribosomes revealed that the amount of cytosolic polysomes is lower and that of monosomes is higher in cold-acclimating plants compared to control plants at both time points (Figure 2A;  Supplemental Figure S4A). This finding was in line with previous data obtained from cold-acclimating Arabidopsis (*Arabidopsis thaliana*) plants (Beine-Golovchuk et al., 2018). Our data suggest a global cold-induced reduction of cytosolic translation initiation activity by ∼30% throughout the analyzed acclimation time course (Figure 2D;  Supplemental Figure S4D). We detected a similar reduction in the ratio of chloroplast polysomes to monosomes, as determined by RNA gel blot analyses of the gradient distribution for the plastid 16S rRNA at both acclimation time points (Figure 2, B–D; Supplemental Figure S4, B–D). Relative rRNA accumulation (as a measure of ribosome quantity), however, did not change significantly (Figure 3), suggesting an overall stable ribosome pool during cold acclimation.

In sum, we observed a similar but overall moderate global effect of low temperature on both chloroplast and cytosolic translation initiation levels.

### Extensive cold-induced translational regulation of specific chloroplast transcripts

**Figure 4:**
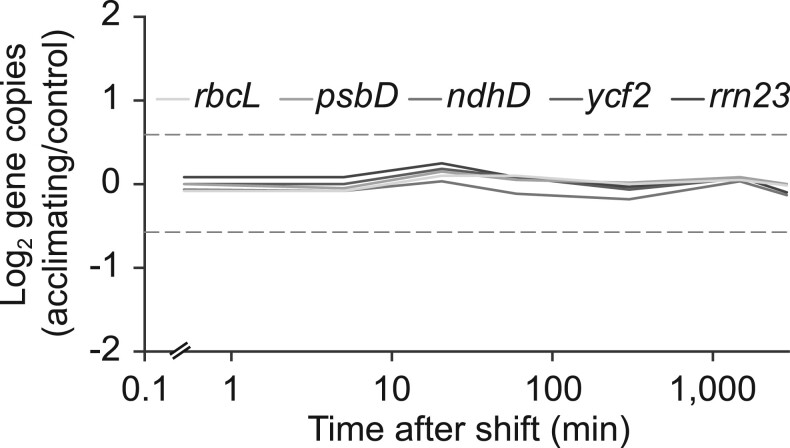
Constant chloroplast genome copy numbers in tobacco chloroplasts during acclimation to low temperature. Log_2_-transformed fold relative changes in chloroplast genome copy numbers measured by qPCR for the indicated chloroplast genes that are located in representative genomic regions at the designated time points after the temperature shift. Dashed horizontal lines, 1.5-fold change. Note the logarithmic scale of the *x*-axis. Results are derived from six individual plants used as independent biological replicates (single plots for individual genes are shown in Supplemental Figure S5).

Most of the core subunits of the photosynthetic machinery are plastid-encoded and chloroplast gene expression responds to environmental stimuli such as light (Zoschke and Bock, 2018). A possible contributor to the regulation of chloroplast gene expression may be the ploidy level of the chloroplast genome, which is known to vary, for example, in response to developmental cues (Sakamoto and Takami, 2018). However, quantitative PCR (qPCR) analysis of representative regions of the chloroplast genome failed to detect significant changes in chloroplast genome copy number in response to low temperature (Figure 4;  Supplemental Figure S5). This finding indicated that adjustments in chloroplast ploidy levels do not substantially contribute to cold acclimation.

**Figure 5:**
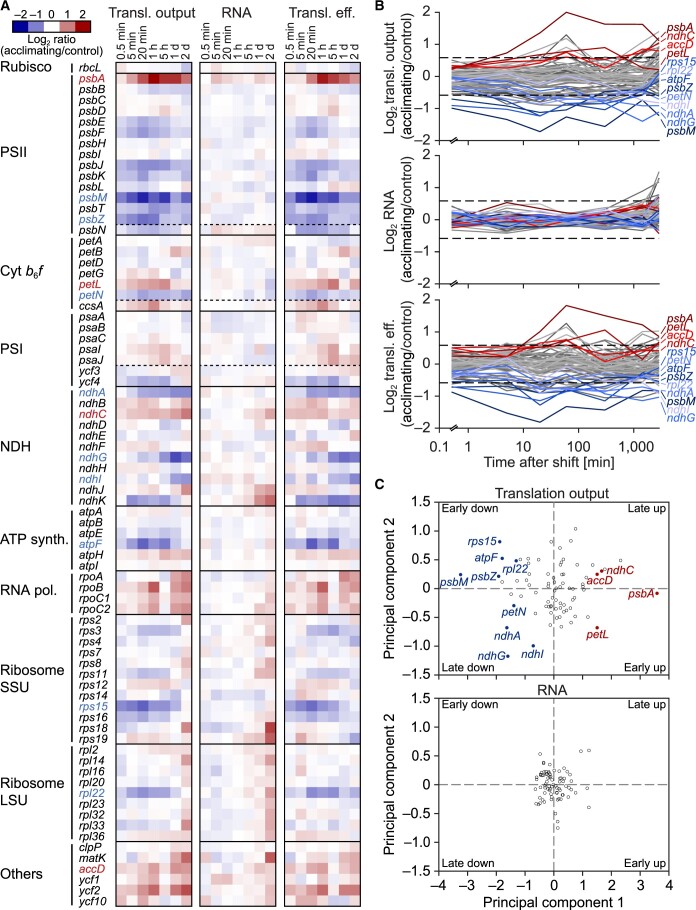
Extensive translational regulation in tobacco chloroplasts during acclimation to low temperature. A and B, Heatmaps and line plots representing the Log_2_-transformed fold-changes in translation output (transl. output), transcript accumulation (RNA), and translation efficiency (transl. eff.) for all chloroplast protein-coding genes at the indicated time points after the temperature shift. Upregulated and downregulated genes, whose translation output changed >1.5-fold (∼10% highest and lowest fold changes) with statistical significance (*q*-value < 0.1) for at least three time points (in total) or two consecutive time points, were considered as cold-responsive and labeled with red and blue letters, respectively. For each time point, data are based on results obtained from three individual plants used as independent biological replicates (in Supplemental Data Set 3). In A, red, upregulation; blue, downregulation, as indicated by the color scale. Genes are grouped according to the following functional assignments: Rubisco, PSII, cytochrome *b*_6_*f* complex (cyt *b*_6_*f*), PSI, NAD(P)H dehydrogenase-like complex (NDH), ATP synthase (ATP synth.), RNA polymerase (RNA pol.), ribosome small/large subunits (Ribosome SSU/LSU), and others. Dashed horizontal lines separate genes encoding structural components (top) and assembly factors (bottom) of the respective complex. In (B), cold-responsive genes whose translation output is upregulated or downregulated are plotted in different shades of red and blue, respectively. All nonresponsive genes are plotted in different shades of gray. Dashed horizontal lines, 1.5-fold change. Note the logarithmic scale of the *x*-axis. C, Principal component analysis of cold-induced changes in translation output and transcript accumulation of chloroplast genes. Fold-changes in translation output (top) and transcript accumulation (bottom) for all chloroplast protein-coding genes at seven time points after cold shift were used. Cold-responsive genes (≥1.5-fold significant changes for at least two consecutive time points or three time points in total) are marked with filled circles and colored labels (blue, downregulated; red, upregulated). Note that principal component 1 separates genes based on direction of fold-change, while principal component 2 separates genes with different temporal responses (early and late). Note that there are no significant changes for transcript levels.

**Figure 6:**
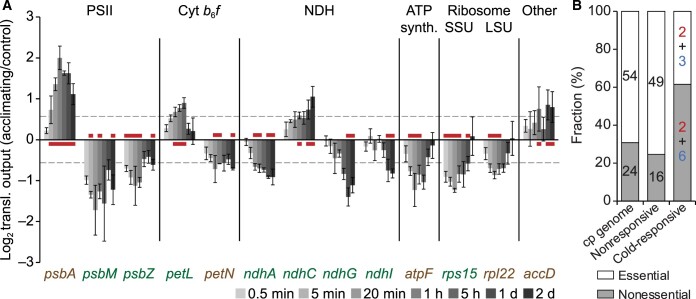
Many cold-responsive genes alter translation output gradually and are not essential for autotrophic growth. A, Bar plot representing the Log_2_-transformed fold-changes in translation output for the 13 cold-responsive genes at the indicated time points after cold shift. Red lines indicate time points with translation output changes >1.5-fold with statistical significance (*q*-value < 0.1). Essential and nonessential genes are labeled in brown and white color, respectively. Vertical lines separate the different protein complexes (abbreviations as in Figure 5A). Error bars denote the standard deviation based on three individual plants used as independent biological replicates. Dashed horizontal lines, 1.5-fold changes. B, Stacked bar plot showing the fraction of nonessential and essential genes in the groups of all protein-coding (“chloroplast genome”), nonresponsive, and cold-responsive chloroplast genes. The respective number of genes is given within the bars (for cold-responsive genes, these numbers are separately shown for genes with increased and decreased translation output and colored in red and blue, respectively). cp genome: chloroplast genome.

To identify possible cold responses in chloroplast gene expression at levels downstream of genome copy numbers (i.e. transcription, transcript accumulation, or translation) in a time- and cost-efficient manner, we applied a microarray-based chloroplast-targeted ribosome profiling approach (Zoschke et al., 2013; Schuster et al., 2020). To this end, we harvested the aerial parts of acclimating and control plants at seven defined time points in a time course starting 30 s and ending 2 days after the cold shift (Figure 1A). For each selected time point, the examined three biological replicates showed high reproducibility (average Pearson’s *R* values were 0.94 for ribosome footprint data and 0.97 for mRNA data; Supplemental Data Set 3). After normalization, we determined average ribosome footprint abundance (as proxy of the translation output) and mRNA abundance (i.e. transcript accumulation) before calculating relative changes during cold acclimation for each chloroplast reading frame. Footprint abundances in a given reading frame reflect translation output, that is, protein synthesis level, because each elongating ribosome leaves one footprint. We defined translation efficiencies as the ratio between ribosome footprint abundance and mRNA abundance, which allowed us to distinguish between translational and transcriptional regulation, both of which can modulate translation output. The ratios of relative changes in chloroplast translation output, transcript accumulation, and translation efficiency between acclimating and control plants are shown as heatmaps and line plots in Figure 5, A and B, respectively, for all chloroplast reading frames at each time point after the temperature shift. These results demonstrated that chloroplast transcript levels do not significantly change during 2 days of cold acclimation (Figure 5, A–C). In contrast, the relative translation output of many chloroplast genes changed gradually in response to low temperature (Figures 5, A–C and 6A). We considered the top and bottom 10% fold-changes of translation output in all analyzed time points as potentially physiologically relevant (corresponding to approximately >1.5-fold changes in either direction). Applying this threshold, we found that 21 and 17 chloroplast genes displayed a higher and lower relative translation output, respectively, for at least one time point after the cold shift, relative to control plants (Figure 5, A and B;  Supplemental Data Set 1). Notably, 13 of these chloroplast genes showed a statistically significant and larger than 1.5-fold change in translation output after the shift for at least two consecutive time points or three time points in total, and were classified as cold-responsive (Figures 5, B and 6, A; details in “Materials and methods” and Supplemental Data Set 3). Three of these genes, *psbA*, *psbM*, and *psbZ*, encode PSII subunits, whose translation output is altered in opposite directions. *psbA* translation was rapidly and durably induced in response to low temperature, with a maximum induction of four-fold, which was the strongest observed translational activation of all genes in the genome (Figures 5, A, B, and Figures 5, A, B and 6, A). In contrast, *psbM* translation substantially decreased throughout the entire time course and exhibited the strongest reduction in translation output of all chloroplast genes (greater than three-fold; Figures 5, A, B, and 6, A). We observed a slightly less pronounced decrease for *psbZ* translation output (Figures 5, B and 6, A). We also noticed opposite directions of regulation for subunits residing in the same protein complex for the cyt *b*_6_*f* and NAD(P)H dehydrogenase-like (NDH) complexes (with translation output of *petL* and *ndhC* increasing, and that of *petN*, *ndhA*, *ndhG*, and *ndhI* decreasing; Figures 5, B and 6, A). Besides *psbA*, *petL* displayed the most consistent cold-induced translational stimulation (significantly increased translation output over three consecutive time points; Figures 5, B and 6, A). Only two additional genes showed a cold-induced promotion of translation, *ndhC* and *accD* (the latter encoding an acetyl-CoA carboxylase subunit; Figures 5, B and 6, A). In addition to the PSII genes *psbM* and *psbZ*, the translation output of many genes decreased in response to cold, including *atpF*, *ndhA*, *ndhG*, *ndhI*, *petN*, *rps15*, and *rpl22*, which encode subunits of the ATP synthase, NDH and cyt *b*_6_*f* complexes, and the chloroplast ribosome, respectively (Figures 5, B and 6, A). Most of these cold-responsive genes code for subunits of photosynthetic complexes, with NDH and PSII genes being particularly overrepresented. Interestingly, many of the cold-responsive genes are not essential for autotrophic growth at moderate temperature (Scharff and Bock, 2014). In fact, nonessential genes were strongly overrepresented within the group of cold-responsive genes (Figure 6B), possibly suggesting a specific contribution to cold acclimation.

**Figure 7:**
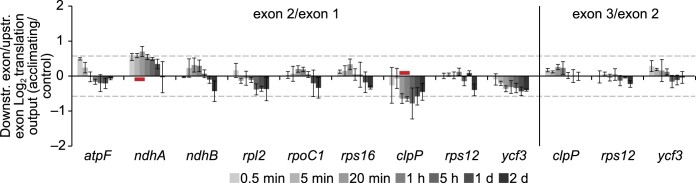
Ribosome coverage in exons of intron-containing genes during cold acclimation. Bar plot showing the comparisons of Log_2_-transformed fold changes in ribosome footprint abundances of individual exons for nine indicated intron-containing reading frames at the specified time points after cold shift (ratios of exon 2/exon 1 and exon 3/exon 2 on the left and the right side, respectively; downstr. = downstream, upstr. = upstream), giving a measure of relative splicing efficiencies during acclimation to low temperature. The three intron-containing genes *petB*, *petD*, and *rpl16* were excluded from the analysis due to the extremely small size of their first exons (6–9 nt), which did not allow the reliable examination of translation output. The vertical line separates results of exon 2/exon 1 and exon 3/exon 2 comparisons. Red lines indicate time points with changes >1.5-fold with statistical significance (*q*-value < 0.1). Error bars denote the standard deviation of results obtained from three individual plants used as independent biological replicates. Dashed horizontal lines, 1.5-fold changes.

Given the observed lower translation output of the intron-containing genes *atpF* and *ndhA* upon cold shift (Figures 5, A, B, and 6, A), and the known effects of higher temperature on chloroplast group II intron splicing (Karcher and Bock, 2002), we wondered whether low temperature might influence splicing efficiency. If true, we would expect a lower ribosome footprint abundance in downstream exons compared to upstream exons (Zoschke et al., 2013; Zoschke and Bock, 2018). The analysis of ribosome footprint abundances in individual exons did not reveal any significant cold-induced splicing defects for *atpF* and most other intron-containing reading frames (Figure 7;  Supplemental Data Set 3). Yet, the splicing efficiency of *ndhA* did substantially (>1.5-fold) and significantly increase, and that of *clpP* (intron 1) decreased in the cold (Figure 7). However, considering the direction of changes (with higher and lower splicing efficiencies, but inverse and virtually no changes in translation output for *ndhA* and *clpP*, respectively), the observed effects of low temperature on splicing could not account for the observed cold-induced changes in translation output of these genes (Figures 7, 5, A and B).

Overall, we observed no significant changes in chloroplast genome copy number or transcript accumulation in response to low temperature (Figures 4, 5, A and B). We concluded that the dynamic changes in translation output must be predominantly caused by altered translation efficiencies (Figure 5A and B). Taken together, our results show that (1) translational adjustments drive cold responses in chloroplast gene expression and (2) low temperature especially triggers translational regulation of nonessential genes.

### Low temperature causes locally altered ribosome distribution on chloroplast reading frames

**Figure 8:**
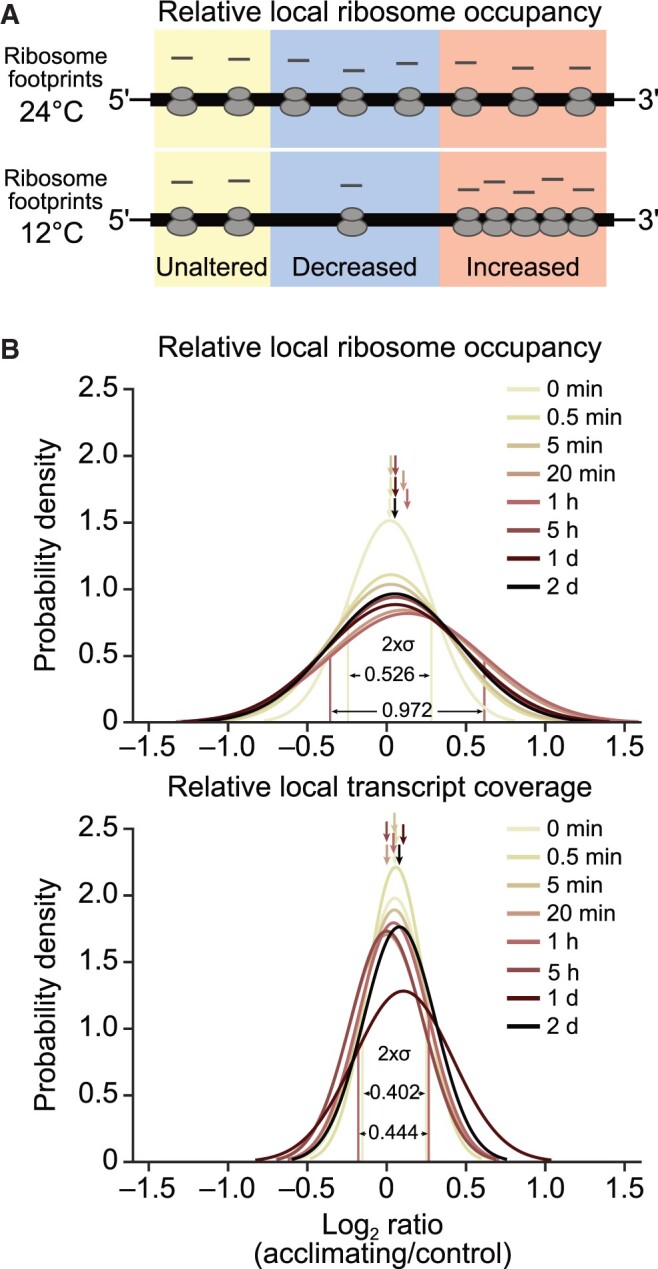
Locally altered distribution of chloroplast ribosomes during acclimation to low temperature. A, Schematic diagram showing hypothetical cold-induced local changes in relative ribosome occupancy (i.e. ribosome redistribution) of a reading frame (thick horizontal line) whose overall translation output is unaltered after cold shift (thin lines depict untranslated regions). Regions with locally unaltered, decreased and increased relative ribosome occupancy are shaded in yellow, blue, and red, respectively. Note that the overall ribosome loading of the reading frame is unaltered. B, Probability density plots of locally altered relative ribosome occupancies (upper) and virtually unaltered relative local transcript coverage (lower) during cold acclimation. For each probe in protein-coding regions, relative local ribosome occupancy and transcript coverage were determined as described previously (Schuster et al., 2020), respectively, and a ratio calculated between acclimating and control plants for each acclimation time point. The distribution of these ratios was visualized in probability density plots, which show the estimated Gaussian distribution of log_2_-transformed fold changes at the indicated time points after shift. The region of [–σ,σ] (representing 68.27% of the area under the density curve, σ: standard deviation) is labeled for relative local ribosome occupancy and transcript coverage at 1-h time point (displaying maximum change of local ribosome distribution after cold shift) and 0-min control. Average distribution numbers are marked by arrows and are shown with σ values in Supplemental Table S1. Results are based on three independently analyzed individual plants as biological replicates. Note that the 1-day time point of relative local transcript coverage showed slightly lower reproducibility (i.e. higher median standard deviation of biological replicates) possibly explaining its slightly different distribution.

The regulation of translation usually occurs at the level of translation initiation (Hershey et al., 2012), which is easily detectable by ribosome profiling due to the influence of translation initiation on the abundances of ribosome footprints throughout reading frames (Figure 5). However, regulation of co-translational events (e.g. protein folding, localization, cofactor binding, and protein complex assembly) occurs by adjustments in local translation elongation behavior (Sharma and O’Brien, 2018) and is controlled, among other factors, by mRNA structure (Choi et al., 2018), which in turn is strongly influenced by temperature (e.g. Zhang et al., 2018). Such regulation of translation elongation becomes visible in ribosome profiles as locally altered ribosome occupancy (e.g. Mohammad et al., 2019; Figure 8A), a feature that is independent of reading frame-wide changes in ribosome occupancy (as it is typical for the regulation of translation initiation). We thus compared locally altered ribosome occupancy between cold-acclimating and control tobacco chloroplasts within each reading frame by normalizing ribosome footprint signal intensities for each individual probe within a reading frame to the sum of the footprint signals observed for the respective reading frame and comparing these values for temperature-shifted and control plants, as previously described (Chotewutmontri and Barkan, 2018; Schuster et al., 2020; Supplemental Figure S6). By this method, we can identify locally confined elongation-based alterations in ribosome distribution, regardless of the overall initiation-based change in ribosome footprint abundance of the entire reading frame during acclimation to low temperature. Calculating overall changes in local ribosome distribution (i.e. pausing behavior) revealed an immediately altered translation elongation behavior after exposure to low temperature (Figure 8B). Cold-induced variations in local ribosome distribution reached a maximum change 1 h after the cold shift, before they gradually returned to levels that are more similar to the control (Figure 8B).

Seven chloroplast genes displayed probes with an over two-fold rise in relative ribosome occupancy for at least two consecutive time points with statistical significance, whereas four genes showed lower ribosome occupancy (Supplemental Figure S6). For instance, two probes located in *psbK* and one located in *atpI* showed an increase in relative ribosome occupancy, whereas one probe in *ndhG* exhibited decreased ribosome occupancy (Supplemental Figure 7).

We observed no significant changes when performing the same analyses with our transcriptomic data (Figure 8B;  Supplemental Figure S8), strongly arguing for ribosome redistribution as being responsible for the observed local changes in the ribosome footprint data and excluding locally changed transcript levels that would be caused, for example, by altered mRNA processing. Taken together, although our approach does not have the resolution to narrow down pausing events to specific mRNA sequences (or structures), our results clearly demonstrate cold-induced altered ribosome distribution on chloroplast reading frames that is progressively restored upon prolonged exposure to low temperature.

### During cold acclimation, PetL accumulates nonstoichiometrically to the cyt *b*_6_*f* core complex

**Figure 9:**
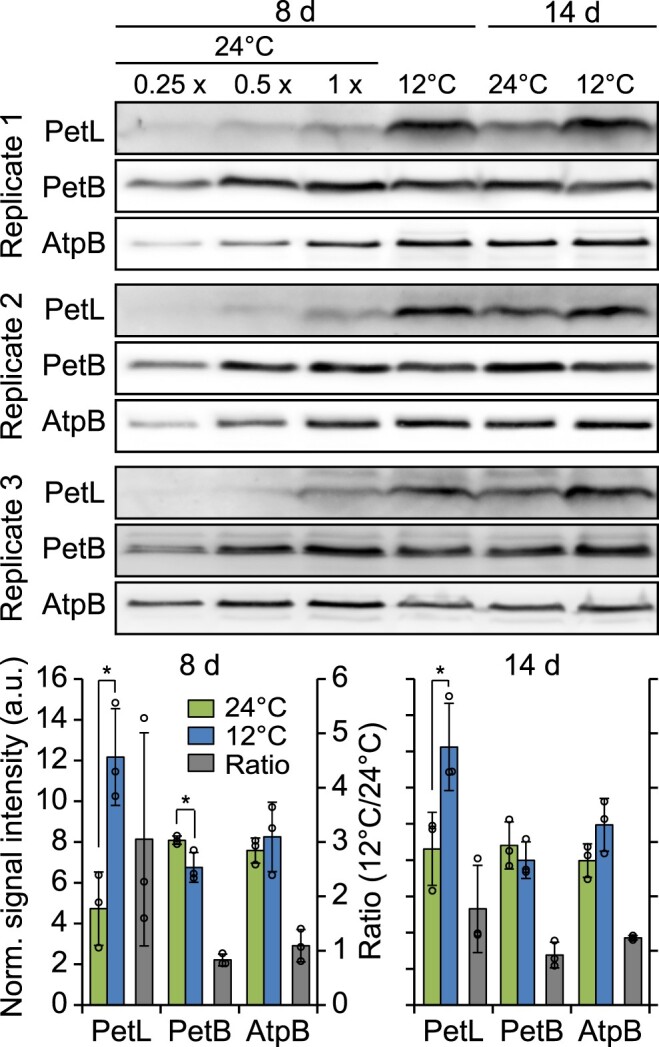
PetL overaccumulates in comparison to the cyt *b*_6_*f* core subunit PetB during acclimation to low temperature. Upper: Immunoblot analyses of PetL, PetB, and AtpB accumulation at the indicated time points after cold shift. One microgram of thylakoid proteins isolated from mature leaves of acclimating (8 or 14 days after shift to 12**°**C) and control plants (grown at 24**°**C to the same developmental stage) were used for immunoblot analyses. In addition, a dilution series is shown for the control plants on the left (dilutions as indicated on top of the blots). Lower: quantification of immunoblot signal intensities. Each blot was normalized to the average signal of all samples for all analyzed proteins (to eliminate technical deviations causing different signal intensities between independent protein membranes and antibodies). Note that this analysis is intended to enable the comparison of signal intensities for a given protein between different conditions but does not allow any comparative assessments for the accumulation of different proteins. Normalized signal intensities were averaged for results obtained from three thylakoid isolations derived from mature leaves of individual plants used as independent biological replicates (left ordinate, green and blue bars for 24**°**C and 12**°**C, respectively). The ratios of signal intensities between 12**°**C and 24**°**C were calculated for each developmental stage and protein (right ordinate, gray bars). The diagrams for plants that were cold-shifted for 8 and 14 days have identical *y*-axes scales. Error bars denote the standard deviation. Asterisks indicate statistically significant differences (*P* < 0.05, two-sided Student’s *t* test, Supplemental Data Set 1). Norm.: normalized; a.u.: arbitrary units.

We identified 13 cold-responsive chloroplast genes, 8 of which are not essential for autotrophic growth (Figure 6). In view of the general reduction in translational activity upon shift to low temperature, translational upregulation in the cold appears particularly challenging to achieve and, therefore, may point to physiological relevance. Indeed, the most upregulated gene was *psbA* (Figure 5, A and B), whose cold-triggered translational enhancement is consistent with greater photodamage occurring at lower temperatures, which in turn leads to an increased demand for PSII repair (see “Discussion”). Considering consistency and extent of the response, the second most upregulated gene was *petL* (Figures 5, A, B, and 6, A). *petL* encodes a small nonessential cyt *b*_6_*f* subunit, whose detailed function is not fully understood (Takahashi et al., 1996; Breyton et al., 1997; Fiebig et al., 2004; Schöttler et al., 2007a; Schwenkert et al., 2007). Based on the cold-induced induced translation of the *petL* mRNA, which is unique among plastid-encoded cyt *b*_6_*f* subunits (Figure 5), we wondered whether PetL accumulates to nonstoichiometric levels relative to the core subunits of the cyt *b*_6_*f* complex after cold exposure. To address this question, we raised a PetL-specific antibody to allow the detection of the protein in thylakoid protein extracts (see “Materials and methods”; Supplemental Figure S9). Immunoblot analyses revealed significantly increased PetL accumulation in the thylakoids of plants that were cold-acclimated for 8 or 14 days in comparison to control plants that were grown at ambient temperature to a similar developmental stage (Figure 9). In contrast, the parallel examination of the accumulation of the essential cyt *b*_6_*f* complex subunit PetB (cyt *b*_6_) showed even slightly reduced or virtually unchanged levels of the cyt *b*_6_*f* core complex during cold acclimation (Figure 9). These data (1) show that the cold-induced promotion of *petL* translation leads to increased PetL protein levels and (2) demonstrate that the cold-induced changes in PetL protein abundance occur independently from changes in cyt *b*_6_*f* core complex accumulation. Together, these data suggest a novel function for PetL during cold acclimation.

### PetL facilitates normal cyt *b*_6_*f* accumulation and is crucial for photosynthetic cold acclimation in mature tobacco leaves

**Figure 10:**
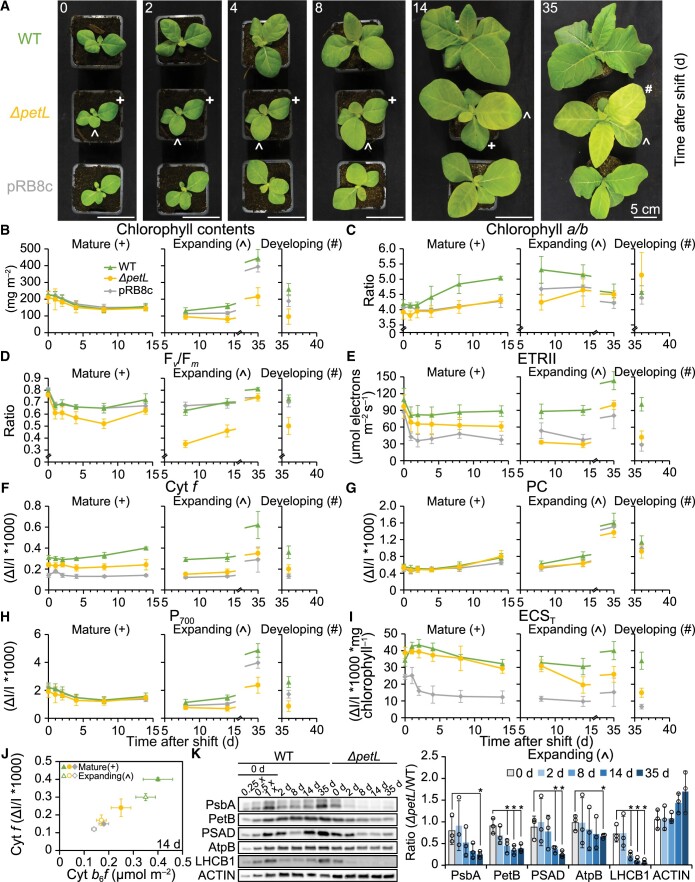
Impaired photosynthetic cold acclimation of the *ΔpetL* mutant. A, Phenotypes of WT, *ΔpetL* and pRB8c plants at the indicated time points after cold shift. These cold-shift experiments were repeated 3 times with at least eight plants, displaying similar cold-induced chlorotic phenotypes for *ΔpetL* mutants. Mature and expanding leaves used for experiments shown in B-K are labeled with crosses and circumflexes, respectively, in the *ΔpetL* mutant. A leaf that fully developed (i.e. newly emerged; referred to as “developing leaf”) in the cold is labeled with a hashtag and was analyzed 36 days after cold shift. B–I, Photosynthetic parameters of mature and expanding leaves of WT (green), *ΔpetL* (yellow) and pRB8c (gray) plants at the indicated time points (d) after cold shift: chlorophyll contents (B), chlorophyll *a/b* ratio (C), maximum quantum efficiency of PSII in the dark-adapted state (F_*v*_/F_*m*_) (D), maximum capacity of linear electron transport (ETRII) (E), in vivo contents of redox-active cyt *f* (F), in vivo contents of redox-active plastocyanin (PC) (G), in vivo accumulation of the PSI reaction center (P_700_) (H), and maximum amplitude of the electrochromic absorption shift (ECS_T_) (I). In (B) and (F–H), data were normalized to leaf area. The data from leaves that expanded in the cold (labeled with circumflex) were separated from data obtained from the developing leaf (leaf that fully developed, that is, newly emerged in the cold) (labeled with hashtag) by vertical dashed gray lines. In (B–I), the statistically significant differences of results are shown in Supplemental Data Set 4. J, Correlation between the maximum difference transmittance signal of cyt *f* with the contents of cyt *b*_6_*f* complex per leaf area in mature and expanding leaves of WT, *ΔpetL* and pRB8c plants at 14 days after cold shift. In (B–J), all in vivo measurements were performed on intact leaves. PSI was quantified using the difference transmission signal of P_700_. cyt *f* serves as a proxy of redox-active cyt *b*_6_*f* complex. ECS_T_ serves as a measure for the light-induced pmf across the thylakoid membrane. Error bars indicate the standard deviation of measurement results from at least seven individual plants used as independent biological replicates for in vivo measurements, and at least three thylakoid isolations each using at least ten individual plants as independent biological replicates for photosynthetic complex quantification (data shown in Table 1). K, Immunoblot analyses of core subunits of major photosynthetic complexes in expanding leaves of WT and *ΔpetL* mutant plants at the indicated time points after cold shift. Error bars indicate the standard deviation of results obtained from three individual plants used as independent biological replicates (quantification details in Supplemental Figure S10). Asterisks indicate statistically significant differences of the ratios (*ΔpetL*/WT) for the indicated time points after cold shift compared with the 0-day control (*P* < 0.05, two-sided Student’s *t* test, Supplemental Data Set 1).

Based on its cold-induced expression and overaccumulation, we hypothesized that the PetL protein contributes to cold acclimation of the photosynthetic apparatus. To address this possibility, we grew a *petL* knockout mutant (Fiebig et al., 2004), a WT control and the cyt *b*_6_*f* knockdown mutant line pRB8c (Loiacono et al., 2019) at 24°C, and then shifted the plants to 12°C for up to 36 days (Figure 10A). Prior to the shift (0 days), both mutants were phenotypically indistinguishable from the WT, as previously reported (Schöttler et al., 2007a; Schwenkert et al., 2007; Loiacono et al., 2019; Figure 10A; leftmost part). Accordingly, directly before cold shift, the first true leaves of the seedlings (which were already fully expanded and subsequently will be referred to as “mature leaves”) exhibited only minor differences in chlorophyll content per leaf area, chlorophyll *a/b* ratio and F_*v*_/F_*m*_ (Figure 10, B–D, left). In addition, the maximum difference transmittance signals (ΔI/I * 1,000) of plastocyanin and P_700_, the latter used as in vivo measure for the content of PSI per leaf area, were very similar (Figure 10, G–H, left panels). Prior to the cold shift, *ΔpetL* and pRB8c plants displayed reduced levels of the maximum light-induced difference transmission signal of cyt *f*, used here as a proxy for the content of the redox-active cyt *b*_6_*f* complex per leaf area, as expected (Figure 10F). Consequently, linear electron transport capacity of PSII (ETRII) decreased moderately (Figure 10E, left), with the effects being somewhat stronger in the pRB8c background. Furthermore, we measured the maximum amplitude of the electrochromic shift signal (ECS) in saturating light (ECS_T_) as a proxy for the light-induced proton motive force (pmf) across the thylakoid membrane (Takizawa et al., 2007). Due to its lower contents of the cyt *b*_6_*f* complex (Figure 10F) and lower capacity of linear electron transport (Figure 10E), the pRB8c mutant line failed to generate a similar maximum pmf across the thylakoid membrane as the WT or the *ΔpetL* mutant (Figure 10I).

After the cold shift, mature leaves could only readjust their preestablished photosynthetic apparatus to the lower temperature. During the first 2 days of cold acclimation, mature leaves of the WT, *ΔpetL* and pRB8c plants displayed a significant decrease in F_*v*_/F_*m*_ (Figures 1, B and 10, D), while chlorophyll contents and chlorophyll *a/b* ratios remained virtually unaltered (Figure 10B and C). The reduction in F_*v*_/F_*m*_ was most pronounced in *ΔpetL* plants (Figure 10D). During extended cold acclimation (between 2 and 14 days), mature leaves of *ΔpetL*, WT and pRB8c showed similar reductions in chlorophyll (Figure 10B) and PSI contents (Figure 10H), while plastocyanin contents slowly increased in all genotypes (Figure 10G). However, only the WT showed a pronounced increase in the chlorophyll *a/b* ratio that is typical of cold acclimation (Schöttler et al., 2017) and indicated a specific degradation of antenna proteins (light-harvesting complex, LHC) attached to PSII, which bind both chlorophyll *a* and *b* (Figure 10C). In both mutants, the chlorophyll contents of mature leaves declined, but the *a/b* ratio remained largely unaltered during cold acclimation, indicating that a parallel degradation of both PSII and its LHC likely occurred. While the WT showed increasing levels of redox-active cyt *f* in cold-exposed mature leaves, this was not the case in the mutants (Figure 10F), thus leading to increasing differences in ETRII (Figure 10E), and a further impairment of thylakoid membrane energization in the pRB8c plants (ECS_T_; Figure 10I).

To confirm the in vivo spectroscopic data and demonstrate that the adjustment of PSII contents in mature leaves of the mutants differs from that in the WT, we performed a detailed characterization of the photosynthetic apparatus 14 days after the cold shift (Table 1). To distinguish between general photosynthetic defects in the mutants and additional cold-induced defects, we compared cold-acclimated plants and plants at a similar developmental stage grown at 24°C (Table 1). In line with previously published data (Schöttler et al., 2007a; Loiacono et al., 2019), most photosynthetic parameters in the mutants were indistinguishable from those in the WT at 24°C. The chlorophyll a/b ratio and content per leaf area, F_*v*_/F_*m*_, ETRII, and the absolute contents of PSII, plastocyanin and PSI on a leaf area basis were virtually identical in all three genotypes (Table 1). Cyt *b*_6_*f* contents per leaf area decreased by 30% in both mutants, relative to the WT. At 12°C, in mature leaves of the WT, PSII and cyt *b*_6_*f* contents were only slightly reduced, relative to plants grown at 24°C, but chlorophyll contents per leaf area and PSI content decreased by almost 50%. In both mutants, the cold-induced reduction in chlorophyll and PSI contents was the same as in the WT, in line with the in vivo changes of the difference transmittance signal of P_700_ (Figure 10H). As suggested by the differences in the chlorophyll *a/b* ratio in cold-acclimating mature leaves, the drop in PSII contents at 12°C was much more pronounced in the two mutants than in the WT. We validated the use of the maximum in vivo difference transmittance signals of cyt *f* as a proxy for the cyt *b*_6_*f* content during the cold acclimation kinetic (Figure 10F) by plotting the cyt *f* signals measured at Day 14 against the absolute contents of cyt *b*_6_*f* (Figure 10J). We obtained a strong correlation between both signals, as expected.

**Table 1:** Absolute quantification of photosynthetic parameters 14 days after cold shift in mature leaves

Genotype and Temperature	Chl. *a/b*	Chl. Contents [mg m^−2^]	F_*v*_/F_*m*_	ETRII [µmol m^−2^ s^−1^]	PSII [µmol m^−2^]	Cyt *b*_6_*f* [µmol m^−2^]	Plastocyanin [µmol m^−2^]	PSI [µmol m^−2^]
**WT 12°C** (*n* = 5)	**5.05** ± 0.06	**158.0** ± 21.3	**0.72** ± 0.03	**91.2** ± 7.5	**0.77** ± 0.10	**0.40** ± 0.06	**1.81** ± 0.20	**0.35** ± 0.05
***ΔpetL* 12°C** (*n* = 4)	**4.31** ± 0.10	**146.0** ± 16.3	**0.63** ± 0.03	**61.3** ± 8.5	**0.54** ± 0.09	**0.25** ± 0.04	**1.75** ± 0.42	**0.32** ± 0.03
**pRB8c 12°C** (*n* = 3)	**4.28** ± 0.14	**153.0** ± 11.8	**0.67** ± 0.02	**37.1** ± 7.3	**0.52** ± 0.06	**0.18** ± 0.02	**1.62** ± 0.21	**0.34** ± 0.04
**WT 24°C** (*n* = 5)	**4.06** ± 0.03	**293.7** ± 13.6	**0.82** ± 0.01	**103.1** ± 7.3	**0.89** ± 0.06	**0.45** ± 0.02	**1.59** ± 0.25	**0.69** ± 0.04
***ΔpetL* 24°C** (*n* = 5)	**3.92** ± 0.08	**284.9** ± 25.7	**0.81** ± 0.01	**95.4** ± 5.8	**0.89** ± 0.08	**0.31** ± 0.05	**1.45** ± 0.15	**0.66** ± 0.06
**pRB8c 24°C** (*n* = 4)	**4.01** ± 0.13	**299.9** ± 30.3	**0.82** ± 0.01	**91.8** ± 5.6	**0.91** ± 0.10	**0.32** ± 0.03	**1.41** ± 0.27	**0.71** ± 0.10

Average values and standard deviation of chlorophyll *a/b* ratio, chlorophyll contents per leaf area, maximum quantum efficiency of PSII in the dark-adapted state (F_*v*_/F_*m*_), maximum capacity of linear electron transport (ETRII) and contents of PSII, cyt *b*_6_*f* complex, plastocyanin and PSI per leaf area in mature leaves of WT, *ΔpetL*, and pRB8c plants 14 days after cold shift. Plants grown at 24°C with comparable developmental stage (similar size) were measured as nonshifted control. The number of biological replicates (*n* = number of independent thylakoid isolations, each pooling mature leaves from at least 10 individual plants) for each condition is specified in the first column together with the genotype and growth temperature.

The statistically significant differences of results are shown in Supplemental Data Set 4.

### During cold acclimation, PetL is crucial for the accumulation of the photosynthetic apparatus in expanding and developing leaves

**Figure 11:**
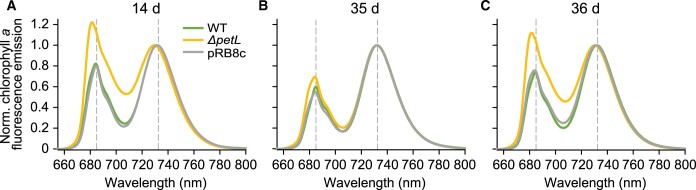
77K chlorophyll *a* fluorescence emission spectra of expanding and developing leaves of WT, *ΔpetL* and pRB8c recorded after cold exposure. 77K chlorophyll *a* fluorescence emission spectra of thylakoids extracted from expanding leaves of WT, *ΔpetL* and pRB8c recorded after 14 (A) and 35 (B) days of cold exposure. C, data for the developing leaf after 36 days in the cold. In tobacco, fluorescence emission of PSII with fully coupled LHCII peaks at 685-nm wavelength (marked with a vertical dashed gray line), and emission of intact PSI-LHCI peaks at 732 nm (marked with a vertical dashed gray line). The shifted emission maximum at 681 nm is indicative of uncoupled LHCII, while the increased fluorescence emission between 700- and 730-nm wavelength arises from uncoupled LHCI. Spectra were normalized to the emission maximum of PSI-LHCI at 732-nm wavelength. Norm.: normalized.

Different from mature leaves, *ΔpetL* leaves that still expanded in the cold (hereafter referred to as “expanding leaves”) became chlorotic, a conspicuous phenotype that is accompanied by mild growth retardation of the mutant plants. Although pRB8c plants also showed growth retardation, they did not exhibit a similar chlorosis (Figure 10A), thus likely excluding lower cyt *b*_6_*f* levels as the cause of the pigment loss. The small sizes of the still expanding leaves made it technically impossible to measure their in vivo photosynthetic parameters at early time points after the cold shift. Therefore, we only analyzed these leaves on Days 8, 14, and 35 after the cold shift, when the previously measured mature leaves were already too senescent to determine their in vivo parameters. Even though expanding leaves of pRB8c showed similar decreases in ETRII (Figure 10E, right), and a somewhat more pronounced drop in cyt *f* (Figure 10F, right) and thylakoid membrane energization (ECS_T_, Figure 10I) than *ΔpetL* at most time points, the chlorophyll and P_700_ contents of the expanding leaves of the *ΔpetL* mutant were significantly lower than in pRB8c at all time points (Figure 10, B and H, right). Furthermore, despite the significantly impaired ETRII and thylakoid membrane energization, we observed no significant differences for *F_v_/F_m_* between the WT and pRB8c, while *F_v_/F_m_* did significantly decrease in *ΔpetL* after eight and 14 days in the cold (Figure 10D). However, by Day 35, *F_v_/F_m_* had recovered to the same level as in pRB8c in expanding *ΔpetL* leaves. Accordingly, on Day 35, 77K chlorophyll *a* fluorescence emission spectra revealed only minor differences between the WT and both mutants (Figure 11): The maximum emission signals of PSII-LHCII at 685 nm and of PSI-LHCI at 732-nm wavelength were unshifted, demonstrating that the LHCs are well coupled to both PSs. On Day 14, however, when *F_v_/F_m_* in *ΔpetL* was still significantly reduced, shifts in the 77K fluorescence emission maxima of expanding leaves indicated substantial accumulation of uncoupled LHCs of both PSII (emitting at the 681-nm wavelength) (Krause and Weis, 1991) and PSI (emitting at wavelengths between 705 and 730 nm; Castelletti et al., 2003; Croce et al., 2004), suggesting that, in addition to P_700_ and chlorophyll contents, PSII accumulation was also compromised in *ΔpetL*.

Finally, 36 days after cold shift, we measured the in vivo parameters for a leaf that had emerged and developed entirely after transfer to the cold (hereafter referred to as “developing leaf”). Again, even though ETRII (Figure 10E, right), thylakoid membrane energization (ECS_T_, Figure 10I, right) and cyt *f* contents per leaf area were more reduced in pRB8c compared to *ΔpetL*, the chlorophyll contents per leaf area, F_*v*_/F_*m*_, and the P_700_ contents were more affected in *ΔpetL* than in pRB8c (and the WT). Similar to the expanding leaves after 14 days, 77K chlorophyll *a* fluorescence emission spectra revealed a strong accumulation of uncoupled LHCs in developing leaves (Figure 11).

As an alternative approach to validate these data, we conducted immunoblot analyses that revealed gradually decreasing levels of cyt *b*_6_*f*, PSI, PSII, and the LHC protein LHCB1 in *ΔpetL* leaves expanding in the cold (Figure 10K;  Supplemental Figure S10). This result was in line with the developmentally increasing chlorotic phenotype and the impaired photosynthetic parameters of leaves that undergo expansion in the cold (Figure 10, A–I).

It was previously reported that in tobacco the L-subunit plays a role in cyt *b*_6_*f* complex dimer stability based on native gels and sucrose density gradients, in which the monomer was the dominant form of the cyt *b*_6_*f* complex in the *ΔpetL* mutant, while the dimer was predominant in the WT (Schwenkert et al., 2007). To investigate if the impaired dimer formation and stability of the *ΔpetL* mutant was affected by growth temperature, we compared the ratio of dimer to monomer between the WT and the *ΔpetL* mutant grown either at 24°C or at 12°C (Supplemental Figure S11A). In accordance with the previous report, native gel separations followed by immunoblots with PetB antiserum revealed that in plants grown at 24°C, almost all cyt *b_6_f* complex is present as a monomer in *ΔpetL*, while the dimer was clearly detectable in the WT. This effect was temperature-independent, because also in plants grown at 12°C, we hardly detected any dimer in the *ΔpetL* mutant, while the WT accumulated a prominent fraction of the cyt *b_6_f* complex in its dimeric form.

Taken together, our results demonstrate a crucial role for PetL in the acclimation of photosynthesis to low temperature. In mature leaves, cold acclimation of both *ΔpetL* and pRB8c is compromised to a similar extent, pointing to a potential critical role for cyt *b*_6_*f* complex contents. Remarkably, however, in expanding and developing leaves, this newly discovered role of PetL is independent of its function in the accumulation of adequate cyt *b*_6_*f* complex levels, as illustrated by major differences in photosynthetic cold responses of *ΔpetL* and pRB8c mutants, while at the same time, cyt *b*_6_*f* complex contents are similarly lower in both mutants.

## Discussion

Chloroplasts play a central role in perceiving and integrating temperature stimuli in plants (Crosatti et al., 2013; Kleine et al., 2021). Our study sheds light on the essential cold acclimation function of chloroplast translation, reveals crucial principles that mediate chilling responses, and identifies nonessential photosynthesis genes as novel important factors in cold acclimation.

### Translational regulation as key determinant of cold-triggered dynamics in chloroplast gene expression

Our genome-wide, time-resolved analysis of chloroplast gene expression during cold acclimation revealed manifold translational adjustments of specific transcripts (Figure 5). Together with virtually unaltered chloroplast genome copy numbers and transcript accumulation in response to low temperature, these results disclose that cold-triggered regulation of chloroplast gene expression occurs predominantly at the level of translation (Figures 4,Figure 5, A and B). The accumulation of a few transcripts was very mildly altered after 2 days at low temperature (Figure 5, A and B). Hence, regulation of transcription and/or RNA stability may be involved in long-term cold responses as described in Arabidopsis (Kupsch et al., 2012). Cold-responsive translational adjustments occurred mostly within 5 h and then gradually declined (Figure 5, A and B), suggesting the realization of a new steady state in chloroplast gene expression. However, a 2-day cold exposure did not substantially alter photosynthetic complex accumulation in mature leaves, consistent with high complex stability (Krantz et al., 2021; Figure 10, F and H). Therefore, in mature leaves (the predominant tissues used for our gene expression analyses), cold-induced changes in translation output may counterbalance temperature-induced effects of altered stability of specific subunits whose translation output and turnover have likely been evolutionarily balanced for growth at moderate temperature (Zoschke and Bock, 2018; e.g. PsbA/D1 in PSII; see below). Alternatively, specialized temperature-dependent functions of specific chloroplast-encoded proteins may require their cold-induced translational activation or repression (see below).

Cold-induced decreases of translation output can occur very rapidly (e.g. within 30 s for *psbM*, *psbZ*, and *rps15*; Figure 6A), suggesting the operation of sensitive regulatory responses (especially if one considers that the temperature shift is not completed at this time; Supplemental Figure S1). In contrast, the fastest increase of translation output took place only after 5 min (*psbA*; Figure 6A). This temporal difference between the fastest increase and decrease of translation output may be related to different kinetics of translation initiation and elongation (Shah et al., 2013). If cold triggers a lower initiation rate, for example, by release of translation-activating RNA binding proteins, elongating ribosomes would run off the transcript within seconds (even at decelerated elongation rates). However, initiation has slower kinetics than elongation (Szavits-Nossan and Ciandrini, 2020), and binding of cold-induced translational activators may need longer, because their expression or activation requires time and mRNA cis-elements may be occluded by cold-stabilized RNA structures (Neupert et al., 2008). The identification of cold-regulatory trans-factors and cis-elements and their molecular interaction will show if these hypotheses hold true. A possible entry point may come from the recently described cold-induced expression of the nuclear *PROTON GRADIENT REGULATION 3* gene (Nagano et al., 2019) that encodes a translational activator of *petL* (Cai et al., 2011; Rojas et al., 2018), a translationally cold-induced chloroplast reading frame shown in this work to be crucial for photosynthetic cold acclimation (Figure 6).

The identified ribosome redistribution indicated cold-induced locally altered translation elongation behavior (Figure 8B;  Supplemental Figure S6). Programmed ribosome pausing can enable efficient processing of the nascent peptide (Gawroński et al., 2018; Zoschke and Bock, 2018), and its cold-induced alteration may have implications for co-translational mechanisms. Indeed, cold-triggered changes in PsbA elongation have been suggested to impair PSII repair (Grennan and Ort, 2007), an effect that is in line with the rapidly decreased F_*v*_/F_*m*_ values we observed after the cold shift (Figures 1, B and 10, D).

### Global effects of low temperature on chloroplast gene expression

Cold induces multilayered global effects on gene expression, which we verified in chloroplasts. In bacteria, cold shock blocks translation initiation (Weber and Marahiel, 2003). In tobacco, more physiological cold acclimation conditions triggered only mild overall decreases in chloroplast and cytosolic translation initiation, and we observed no stress-induced disomes (Figure 2;  Supplemental Figure S4). This result may reflect the habitual cold exposure of plants, and the evolutionary pressure to sustain growth at low temperatures.

Despite lower ribosome loading of many chloroplast mRNAs (Figures 2, 5, A and B; Supplemental Figure S4), transcript levels remained largely unaltered (Figure 5, A and B). This observation confirms that untranslated or weakly translated chloroplast transcripts are stable in vascular plants, and that translating ribosomes are not required to protect plastid transcripts from degradation (Zoschke and Bock, 2018). Chloroplast ribonucleoproteins (RNPs) stabilize numerous mRNAs at low temperatures (Kupsch et al., 2012) and may also protect ribosome-free transcripts from ribonucleolytic attack during cold acclimation.

Cold should increase ribosome dwell times at stabilized RNA structures. Indeed, chloroplast ribosomes locally redistributed, indicating both increased and decreased pausing (Figure 8, A and B; Supplemental Figure S6). If ribosome availability is limiting, locally increased ribosome loading at one position should induce decreased loading at other locations, and these effects should be almost balanced, an assumption that is supported by our results (Figure 8B). The overall limited extent of ribosome redistribution detected in our analyses may be due to the strong RNA helicase activity of 70S ribosomes (Takyar et al., 2005), which is likely capable of resolving most cold-stabilized RNA structures. In mid- and long-term cold acclimation, ribosome redistribution decreased (Figure 8B), possibly due to the increasing activity of RNA chaperones (Weber and Marahiel, 2003).

The anticipated cold-induced reduction in translation elongation is much stronger (50%–66%; Guillaume-Schöpfer et al., 2020) than that in initiation (∼30%; Figure 2). Why then do ribosomes not pile up downstream of start codons (Supplemental Figure S7)? This is an unlikely scenario for two reasons. First, lower elongation rates can only affect the overall ribosome loading of a given reading frame, if they override the much stronger limitations in initiation (Shah et al., 2013). Second, the number of ribosomes is usually limiting (Shah et al., 2013), thus causing competition between transcripts for available ribosomes. Hence, overall slower elongation causes reduced termination, and fewer ribosomes become available for initiation. In this situation, ribosome loading is unaltered and reduced elongation rates globally limit protein synthesis, which is not detectable in ribosome profiling or polysome experiments. A globally slower elongation rate may be determined by protein pulse labeling or in translational run-off kinetics of ribosome profiling data after inhibiting early elongation steps with lincomycin (Chotewutmontri and Barkan, 2018). However, these data may not be very revealing, because cold-induced slow-down likely affects all mRNAs proportionally to their expression levels and, consequently, the ratios of synthesized proteins will stay constant.

### Low temperature versus high light: similar effects on photosynthesis, divergent responses in chloroplast gene expression

Exposure of plants to low temperatures or high light results in similar imbalances between the light reactions of photosynthesis and the CBB cycle with comparable consequences for metabolism and redox homoeostasis (Huner et al., 1998, 2013). We demonstrated that in both conditions, excitation pressure on PSII and photoinhibition increase (Schuster et al., 2020; Figures 1, B and 10, D) and, consequently, PSII repair (i.e. replacement of photodamaged PsbA and activation of *psbA* translation) is rapidly triggered (Schuster et al., 2020; Figure 5, A, B, and 6, A). Additional acclimation responses buffer PSII damage within 2 days, likely leading to dampened activation of *psbA* translation (Schuster et al., 2020; Figure 5, A, B, 6, A). These responses include light-avoidance movement of chloroplasts (Fujii et al., 2017; Kasahara et al., 2002; Figure 1C;  Supplemental Figure S2), reduction of PSII antenna size (Schuster et al., 2020; Figure 10C), adjustments in photosynthetic complex stoichiometries (Schöttler et al., 2017), thylakoid membrane rearrangements (Kratsch and Wise, 2000), and recovery of electron transport (Stitt and Hurry, 2002).

Apart from the promotion of *psbA* translation, chloroplast gene expression is almost completely differently regulated during high-light acclimation versus cold acclimation. High light exposure or a dark-to-light shift did not induce substantial changes in the expression of specific chloroplast genes (except for translational activation of *psbA*), including translation initiation and ribosome distribution (Chotewutmontri and Barkan, 2018; Schuster et al., 2020). In contrast, low temperature caused massive translational adjustments at both the initiation level and the elongation behavior (Figures 5, A, B, and 8, B; Supplemental Figure S6). Redox signals were suggested to trigger changes in chloroplast gene expression (Allen, 2015). However, the similarity of cold- and high-light-induced redox imbalances and the concomitant regulatory discrepancy in chloroplast gene expression suggest that redox signals are not involved in the observed regulatory responses in chloroplast translation. Only *psbA* translation is induced in both high-light and cold acclimation responses (Schuster et al., 2020; Figure 5, A and B). However, recent data strongly suggest that *psbA* translation is triggered by PsbA photodamage and/or PSII assembly-dependent signals (Chotewutmontri and Barkan, 2020), a model that sufficiently explains the rapid promotion of *psbA* translation in both high light and low temperature.

### Nonessential photosynthetic subunits as novel players in cold acclimation

Most cold-responsive genes are not essential for autotrophic growth at moderate temperature (Figure 6B), suggesting specific roles during cold acclimation. The second most pronounced cold-induced gene (after *psbA*) was *petL* (Figures 5, A, B, 6, A), encoding a nonessential cyt *b*_6_*f* subunit (Takahashi et al., 1996; Breyton et al., 1997). PetL comprises one transmembrane helix, is located at the periphery of the cyt *b*_6_*f* complex (Malone et al., 2019), and facilitates dimer integrity (Breyton et al., 1997; Schwenkert et al., 2007) and developmental stability (Schöttler et al., 2007a) of the cyt *b*_6_*f* complex. Our data demonstrate that PetL overaccumulates during cold acclimation in comparison to the cyt *b*_6_*f* complex core subunit PetB, a finding that suggests a cold-specific function for PetL during acclimation to low temperatures. Furthermore, we found that the *ΔpetL* mutant displays cold-induced chlorosis and photosynthetic defects in expanding and developing leaves (Figure 10), uncovering a visible phenotype for the *petL* knockout in vascular plants, an effect that had previously gone undetected. In *ΔpetL*, besides lower chlorophyll contents, PSI contents per leaf area and F_*v*_/F_*m*_ significantly decreased. The cold-induced defects in *ΔpetL* are not explainable by lower cyt *b*_6_*f* levels, because the cyt *b*_6_*f* knockdown mutant pRB8c, which displays a similarly diminished cyt *b*_6_*f* complex accumulation and, consequently, a comparably impaired ETRII and thylakoid membrane energization, did not become chlorotic and displayed WT-like F_*v*_/F_*m*_ (Figure 10, A, D, and F).

Structural und functional data suggest that the cyt *b*_6_*f* complex can only be active in its dimeric form in vivo, especially because each Rieske iron sulfur protein (PHOTOSYNTHETIC ELECTRON TRANSFER C (PETC)) spans the dimer, being anchored to its transmembrane helix in one monomer, but interacting with the quinol oxidizing site of the second monomer via its hydrophobic domains binding the 2Fe-2S cluster (e.g. Agarwal et al., 2015; reviewed by Sarewicz et al., 2021). Hence, the previously shown impaired cyt *b*_6_*f* complex dimer integrity in the *ΔpetL* mutant (Schwenkert et al., 2007) likely reflects a lower in vitro stability of the dimer in the absence of PetL (potentially caused by increased in vitro dimer sensitivity, e.g. to ions and/or detergent the complex dimer is exposed to during isolation or native gel electrophoresis). This cyt *b*_6_*f* dimer instability cannot mirror the in vivo situation, because an in vivo monomerization of the cyt *b*_6_*f* complex would induce a strong photosynthetic deficiency, which is inconsistent with the observed electron transport rates in the mutant. Furthermore, we showed here that these previously described effects of PetL deficiency on the in vitro cyt *b*_6_*f* dimer stability are temperature-independent (Supplemental Figure S11A). Additionally, our data demonstrate that the cyt *b*_6_*f* complex accumulation correlates well with its in vivo activity under all analyzed conditions (i.e. the slope between redox-active cyt *f* and ETTRII was unaltered in the mutants compared to the WT; Supplemental Figure S11B). Consequently, we conclude that in vivo, no prominent dimer disassembly occurs in the absence of PetL in the conditions we analyzed. Accordingly, a substantial in vivo monomer accumulation caused by cyt *b_6_f* dimer disassembly in the *ΔpetL* mutant can be excluded. Altogether, it thus appears likely that during cold acclimation, PetL acts, at least in expanding and developing leaves, functionally independently of the photosynthetic activity of the cyt *b*_6_*f* complex and either directly influences photosynthetic performance or signaling from the electron transport chain during cold acclimation, a function that may be either linked to or independent of the cyt *b*_6_*f* complex.

In mature leaves, which can only readjust their preestablished photosynthetic apparatus to low temperature, the main difference between the WT and both cyt *b*_6_*f* mutants is their failure to specifically downregulate their LHC contents, relative to the PSII reaction center. In consequence, while PSII contents remained largely unaltered in cold-acclimating WT plants and the chlorophyll *a/b* ratio strongly increased, in both mutants, the chlorophyll *a/b* ratio only mildly increased and PSII contents decreased by ∼40% relative to plants kept at 24°C (Table 1). This differential behavior of the nucleus-encoded LHC points to defects in retrograde signaling from the chloroplast to the nucleus in the mutants (Wu and Bock, 2021). Similar photosynthetic responses during cold and high-light acclimation of WT tobacco might suggest similar underlying retrograde signals, the identity of which remains to be determined. For high-light acclimation, a key role of the redox poise of the plastoquinone pool in regulating LHCII expression was established (Escoubas et al., 1995). The redox state of the plastoquinone pool is transduced by the redox-regulated thylakoid protein kinase STATE TRANSITION 7 (STN7), which is also involved in state transitions (Bellafiore et al., 2005; i.e. LHCII redistribution between PSI and PSII). Mutants deficient in the STN7 kinase are also impaired in the redox-dependent long-term response of the photosynthetic apparatus to changing light qualities (Bellafiore et al., 2005; Dietzel et al., 2011; Goldschmidt-Clermont and Bassi, 2015). STN7 activation is dependent on its binding to the cyt *b*_6_*f* complex (Shapiguzov et al., 2016). Although it is currently unknown where exactly STN7 binds and whether PetL is involved (Dumas et al., 2017), decreased LHCII phosphorylation levels in *ΔpetL* may suggest a regulatory connection between PetL and STN7 (Schwenkert et al., 2007). Potential regulatory links between PetL, the cyt *b*_6_*f* complex, and STN7-dependent phosphorylation, can be investigated in future studies by examining (1) STN7 activity and cyt *b*_6_*f* association in the *ΔpetL* mutant and (2) cold acclimation in *stn7* mutants.

*psbM* and *psbZ* encode small nonessential PSII subunits whose translation output is significantly lower at low temperature (Figures 5, A, B, and 6, A), a response that coincides with the activation of *psbA* translation (Figures 5, A, B, and 6, A). PsbM connects two PSII monomers at the center of the dimer (van Bezouwen et al., 2017), a function that is essential for PSII dimerization in cyanobacteria (Kawakami et al., 2011), but not in plants (Umate et al., 2007). PsbZ is located at the periphery of PSII and connects LHCII to PSII supercomplexes (Swiatek et al., 2001). For PSII repair, LHCII is removed from supercomplexes and PSII dimers monomerize (Theis and Schroda, 2016). Consequently, repression of *psbM* and *psbZ* translation may facilitate efficient PSII repair during cold acclimation. Additionally, low temperature induces decreased LHCII levels (Figure 10K), a response that may be promoted by *psbZ* downregulation. Investigation of cold acclimation in *psbM* and *psbZ* knockout mutants and overexpression lines should reveal the cold-related functions of these genes.

In summary, our data reported here reveal ample effects of low temperature on chloroplast translation that are distinct from high-light responses. We identified nonessential chloroplast genes as novel players in plant cold acclimation, thus opening interesting new lines of research toward (1) uncovering the functions of these genes in cold acclimation; (2) isolating the cis- and trans-acting factors that mediate the cold response; and (3) elucidating the underlying molecular mechanisms of regulation. Ultimately, these efforts will contribute to the generation of new crop plant varieties that can grow under adverse conditions and can withstand the challenges of climate change.

## Materials and methods

### Plant material and growth conditions

For chloroplast gene expression analyses, WT tobacco (*N.* *tabacum*, cv. Petit Havana) was germinated and grown as described before (Schuster et al., 2020). Two identical plant growth chambers (Conviron BDR16, Canada, equipped with Venture 60260 400 W bulbs) with identical growth parameters (in day–night cycles of 16-h light [350 µmol m^−2^ s^−1^] with 60% relative humidity and 8-h dark with 55% relative humidity), apart from temperature, were used for temperature-shift experiments with tobacco seedlings in the four-true-leaf stage. Right before the temperature shift (5 h after start of the photoperiod 21 days after sowing), the aerial part of some plants was harvested and snap-frozen in liquid nitrogen for later use as 0 days control sample. Half of the tobacco plants were shifted from moderate temperature conditions (day/night temperature: 24°C/20°C) to low temperature conditions (day/night temperature: 12°C/12°C, referred to as acclimating plants), whereas the other half of the plants was transferred back to moderate temperature conditions (referred to as control plants) analogously to previously described light shift experiments (Schuster et al., 2020). The aerial part of acclimating and control plants was harvested in parallel at defined time points after cold shift (0.5 min, 5 min, 20 min, 1 h, 5 h, 1 day, and 2 days), snap-frozen in liquid nitrogen and stored at –80°C until further use. To examine comparable developmental stages between acclimating and control plants (despite the mild cold-induced growth retardation), the latest analyzed time point was 2 days after cold shift.

For immunoblot analyses of PetL accumulation in the cold (shown in Figure 9) and absolute quantification of photosynthetic parameters (shown in Table 1), WT and *ΔpetL* as well as pRB8c mutant plants were grown and cold-shifted as described above. Plants were grown for up to 14 days in the cold. Plant growth in the cold (12°C) is delayed compared to growth at ambient temperature (24°C) if cold exposure persists longer than 2 days (Figure 1A). Hence, matching developmental stages between cold-exposed and ambient-temperature-grown plants were achieved by choosing plants with similar size and leaf number (i.e. plants grown at 12°C and harvested after 8 and 14 days of cold exposure were compared to plants of similar size and leaf number grown for 3 and 6 days at ambient temperature, respectively).

For immunoblot analyses and in vivo measurements of photosynthetic parameters (shown in Figure 10), the *petL* knockout mutant, a WT control and the cyt *b*_6_*f* knockdown mutant pRB8c were grown and cold-shifted as described above. Since all genotypes displayed highly similar growth at low temperature (i.e. comparable developmental stages), phenotypes and molecular effects could be comparatively monitored over a long time period (up to 36 days).

### Ribosome profiling

The preparation of tobacco ribosome footprints and RNA, labeling with fluorescent dyes, and hybridization to custom microarrays were performed as described previously (Schuster et al., 2020).

Ribosome and transcript profiling data were analyzed as previously described (Schuster et al., 2020) with minor modifications. Briefly, probes with saturated signals were excluded from the analysis. Local background-subtracted probe signals (F635-B635 and F532-B532, respectively, Supplemental Data Set 3) smaller than 100 were considered as background and accordingly set to zero. The signals of all probes covering protein-coding regions were normalized to the average signal of these probes across all datasets from the cold shift experiments. For each biological replicate (i.e. each microarray hybridization), signals of different probes within a chloroplast reading frame from both shifted and nonshifted samples were normalized to their standard deviation (resulting in equal variance for the probe signals within a reading frame for all three biological replicates). This normalization step was intended to minimize technical deviations caused by different labeling and hybridization efficiencies. Normalized signals from different probes within a reading frame were subsequently averaged for shifted and nonshifted samples, respectively. The normalized ribosome footprint and RNA abundances for each chloroplast reading frame were normalized to the values from the 0-day control sample (Schuster et al., 2020) (collected directly before shift) and averaged across the three biological replicates for each time point. Translation efficiencies were calculated for each reading frame as the ratio between ribosome footprint abundance and RNA abundance. Genes with above 1.5-fold changes (approximately top 10% highest and lowest fold-changes in all analyzed time points, respectively) in translation output were considered as cold-responsive. The significance of changes in gene expression was assessed by two-sided Student’s t test. The resulting *P*-values were adjusted for multiple testing according to Storey’s *q*-value method (Storey, 2002). Differential distribution of ribosomes on chloroplast transcripts (i.e. differential pausing) were evaluated as described before (Schuster et al., 2020).

### qPCR

Genomic DNA was extracted from the aerial parts of acclimating and control plants, using the DNeasy Plant Mini kit (Qiagen, Hilden, Germany) according to the manufacturer’s instructions. DNA quantity and quality were examined by agarose gel electrophoresis. qPCR was performed on a LightCycler 480 Instrument II (Roche Diagnostics, Basel, Switzerland). PCR reactions were prepared using the LightCycler 480 SYBR Green I Master Mix (Roche Diagnostics) and contained 0.1 ng of DNA and 5 pmol of each primer (sequences provided in Supplemental Table S2). Three technical replicates per biological replicate were included. Polymearase chain reaction (PCR) cycling was done as follows: 95°C for 10 min, followed by 40 cycles of 95°C for 10 s, 55°C for 20 s, and 72°C for 15 s. The second derivative maximum method was used to determine cycle threshold values, and qPCR efficiencies for each primer pair were determined from DNA serial dilution curves. Melting curve analysis was performed to verify the specificity of amplified products. Relative copy numbers were determined for five chloroplast genes (*ndhD*, *psbD*, *rbcL*, *23S rDNA*, and *ycf2*), representing the major regions of the chloroplast genome. The nuclear 18S rDNA was used for data normalization.

### Polysome and RNA gel blot analyses

Polysome analysis was performed according to a published procedure (Barkan, 1998) with minor modifications: 4 mL of lysate prepared from 300 mg tobacco tissue was loaded onto a 1-mL sucrose cushion (30% [w/v] sucrose, 100-mM KCl, 40-mM Tris acetate, pH 8.0, 15-mM MgCl_2_, 5-mM 2-mercaptoethanol, 100-µg mL^−1^ chloramphenicol, and 100-µg mL^−1^ cycloheximide) and centrifuged for 1.5 h at 4°C and 303,800 *g* to pellet large RNPs containing monosomes and polysomes. Subsequently, mRNAs from this pellet were size-separated according to their ribosome-loading by ultracentrifugation for 4 h at 4°C and 169,000 *g* in previously described sucrose density gradients (Barkan, 1998). RNAs were extracted and purified from sucrose-gradient fractions, resuspended in 50-µL RNase-free water, and aliquots of 12 µL from each fraction were analyzed by RNA gel blotting as previously described (Barkan, 1998).

### Protein extraction and gel blot analyses

Total cellular proteins were isolated from expanding leaves as described (Cahoon et al., 1992). Four micrograms of purified total protein was separated by SDS–PAGE on Mini-Protean TGX gels (Bio-Rad, Hercules, CA, USA), and transferred onto nitrocellulose membranes in a Mini Trans-Blot Electrophoretic Transfer Cell system (Bio-Rad). For PetL detection, thylakoid proteins were isolated as described before (Schöttler et al., 2004), samples corresponding to 1 µg protein were separated by sodium dodecyl sulfate (SDS)–polyacrylamide gel electrophoresis (PAGE) and blotted onto polyvinylidene difluoride membranes. The membranes were then incubated with antibodies against PetB, PSAD, PsbA, LHCB1, and AtpB, obtained from Agrisera (Vännäs, Sweden; product No., working dilution: AS03034, 1:10,000, AS09461, 1:1000, AS10704, 1:10,000, AS01004, 1:2,000, and AS05085, 1:10,000), and ACTIN obtained from Sigma-Aldrich (St Louis, MO, USA; product No.: A0480, 1:5,000). The antibody against PetL was generated by GenScript Biotech (Amsterdam, Netherlands, amino acid sequence selected as antigen: GLSKIRLI). Immunodetection was performed using ECL Plus detection reagents (GE Healthcare, Chicago, IL, USA) and documented with Syngene G:BOX Chemi XT4 (SynOptics). Signal intensities were quantified using Image Lab software (Bio-Rad).

Native-PAGE analysis was performed as described (Järvi et al., 2011) using 20 mg of ground tissue from mature and expanding leaves.

### Microscopic analyses

Chloroplast arrangement in mesophyll cells of control plants (grown at 24°C) and acclimating plants (grown for 2 days at 12°C) was determined by confocal laser-scanning microscopy (TCS SP5, Leica, Wetzlar, Germany). For the detection of chlorophyll fluorescence, the 405-nm Laser was used to excite chlorophyll and the emission collected between 650 and 700 nm.

### Metabolite profiling

The Methyl-tertiary-butyl-ether extraction method was employed for measurements of the primary and secondary metabolites as described previously (Salem et al., 2016, 2017). About 50 mg of frozen and homogenized leaves were used for the extraction. Primary metabolites were measured as described (Lisec et al., 2006; Perez de Souza et al., 2019) and mass tags identified according to the Golm Metabolome Database (Supplemental Data Set 2; Hummel et al., 2013). Secondary metabolite measurements were performed as described (Perez de Souza et al., 2019; Tohge and Fernie, 2010; Tohge et al., 2016). Aliquots of the samples were injected into Waters ACQUITY UPLC (Waters Milford, MA, USA) coupled to a Q-Exactive Orbitrap-focus system (Thermo Finnigan, San Jose, CA, USA) via an electrospray ionization interface (Perez de Souza et al., 2019). Chromatograms were recorded and processed with Xcalibur (version 2.10, Thermo-Fisher, Waltham, MA, USA), ToxID (version 2.1.1; Thermo-Fisher) or the Refiner MS software (version 6.0, Gene-Data, Basel, Switzerland; Hummel et al., 2011). Annotation of secondary metabolites was based on manual curation of mass spectra, authentic standards, and literature search (Supplemental Data Set 2). Peak areas were normalized based on the fresh weight and the internal standard 13C sorbitol. The significance of changes in metabolites was assessed by two-sided Student’s *t* test. The resulting *P*-values were adjusted for multiple testing according to Storey’s *q*-value method (Storey, 2002).

### In vivo measurements of photosynthetic parameters

For better comparability of functional data between control and cold-exposed plants, all in vivo measurements of photosynthetic parameters were performed at 24°C. During the first 8 days after cold shift, chlorophyll *a* fluorescence parameters were determined with the Maxi-version of the Imaging-PAM M-series (Walz, Effeltrich, Germany). After 14 days, plant size precluded measurements in the Imaging-PAM and the fiber optics version of the DUAL PAM-100 (Walz, Effeltrich, Germany) was used. Leaves were dark-adapted for 30 min prior to the measurement. Then, under light-limited conditions, the light intensity was increased in 150-s intervals. Under light-saturated conditions above 500 µE m^−2^ s^−1^, the light intensity was increased each 60 s. ETRII were derived from the quantum yield Y(II) of PSII and corrected for leaf absorptance measured between 400 and 700 nm wavelength using an integrating sphere attached to a V-650 photometer (Jasco GmbH, Pfungstadt, Germany). PSI-related measurements were performed with the plastocyanin-P_700_ version of the Dual-PAM instrument as described (Schöttler et al., 2007b). Plants were preilluminated at growth light intensity for 3 min prior to measurements, to partially activate the CBB cycle and thereby avoid an acceptor-side limitation of PSI during the determination of the maximum amount of redox-active plastocyanin and PSI. Then, the light intensity was increased stepwise as described above for the chlorophyll *a* fluorescence measurements. The fraction of PSI reaction centers limited at the donor side, Y(ND), was determined as described (Schreiber and Klughammer, 2016).

The maximum amplitude of the ECS was used as a measure for the light-induced pmf across the thylakoid membrane. Leaves were preilluminated for 6 min with saturating light (1,295 μE m^−2^ s^−1^) so that photosynthesis was fully activated and ATP synthase activity was not limited by ATP consumption by the CBB cycle. The saturating illumination was interrupted by 15-s intervals of darkness, and the maximum amplitude of the ECS during the first 250 ms of darkness was determined as a measure for the total light-induced pmf across the thylakoid membrane (Takizawa et al., 2007; ECS_T_). ECS_T_ was normalized to the chlorophyll contents per leaf area, which was determined in 80% (v/v) acetone (Porra et al., 1989). The maximum difference transmittance signal of cyt *f* (ΔI/I) was determined in parallel to the ECS measurements. The amplitude of the difference transmittance signal between the fully oxidized state in saturating light and the fully reduced state reached within 500 ms in darkness was used as a measure of total redox-active cyt *b*_6_*f*. All signals were simultaneously measured between 505 and 570 nm wavelength using the KLAS-100 spectrophotometer (Walz, Effeltrich, Germany) and deconvoluted as previously described (Klughammer et al., 1990; Rott et al., 2011).

### Quantification of photosynthetic complexes

PSII, cyt *b*_6_*f* complex and PSI were quantified spectroscopically in isolated thylakoids as described (Schöttler et al., 2004). PSII and cyt *b*_6_*f* complex contents were determined from difference absorbance signals of cyt *b_559_* (PSII) and cyt *f* and *b_6_* (cyt *b_6_f* complex) measured with a V-750 photometer equipped with a head-on photomultiplier (Jasco GmbH, Pfungstadt, Germany). After complete oxidation of all cytochromes by the addition of 1 mM potassium ferricyanid (+III), cyt *f* and the high-potential form of cyt *b*_559_ were reduced by the addition of 5- mM sodium ascorbate, followed by the addition of 10-mM sodium dithionite to reduce the low-potential form of cyt *b*_559_ and the cyt *b_6_*. Difference absorbance spectra were calculated, baseline-corrected and deconvolved as described (Kirchhoff et al., 2002; Lamkemeyer et al., 2006).

Redox-active PSI content was determined as described (Schöttler et al., 2017). Plastocyanin contents, relative to P_700_, were determined in leaves by in vivo difference absorption spectroscopy in the far-red range of the spectrum and then recalculated based on the absolute P_700_ quantification in isolated thylakoids and finally re-normalized to a leaf area basis with the known chlorophyll content per leaf area.

77K chlorophyll *a* fluorescence emission spectra were measured using a F-8300 fluorometer (Jasco GmbH., Pfungstadt, Germany) on freshly isolated thylakoids equivalent to 10-μg chlorophyll mL^−1^. The sample was excited at 430-nm wavelength with a bandwidth of 10 nm, and the emission spectrum was recorded between 650- and 800-nm wavelength in 0.5-nm intervals with a bandwidth of 1 nm.

### Data availability

All data obtained for this study are presented within the main text, the Supplemental Data Sets 1–4 and the supporting information.

### Accession numbers

Tobacco microarray design was based on the GenBank library under accession number Z00044.2. All discussed genes are annotated in the GenBank library accession number Z00044.2.

## Supplemental data 

The following materials are available in the online version of this article.

**Supplemental Figure S1.** Leaf and soil temperature after cold shift.

**Supplemental Figure S2.** Chloroplast movement after cold shift.

**Supplemental Figure S3.** Metabolite changes during cold acclimation.

**Supplemental Figure S4.** Mild global reduction of cytosolic and chloroplast translation initiation after 20 min at low temperature.

**Supplemental Figure S5.** Relative copy numbers of chloroplast genes during cold acclimation.

**Supplemental Figure S6.** Locally altered ribosome distribution in specific chloroplast reading frames during acclimation to low temperature.

**Supplemental Figure S7.** Ribosome redistribution in specific representative chloroplast reading frames during acclimation to low temperature.

**Supplemental Figure S8.** Relative local chloroplast RNA accumulation during acclimation to low temperature.

**Supplemental Figure S9.** Validation of the specificity of the anti-PetL antibody.

**Supplemental Figure S10.** Immunoblot analysis of core photosynthetic proteins in expanding leaves of cold-acclimating WT and *ΔpetL* plants.

**Supplemental Figure S11.** Cyt *b*_6_*f* dimer stability and activity in mature and expanding leaves of WT, *ΔpetL* and pRB8c plants after cold shift.

**Supplemental Table S1.** Mean and standard deviation parameters of probability density plots of local ribosome and RNA distribution during cold acclimation.

**Supplemental Table S2.** Primers used for qPCR.

**Supplemental Table S3.** In vivo contents of redox-active cyt *f* and ETRII in WT, *ΔpetL* and pRB8c plants after cold shift.

**Supplemental Data Set 1.** Student’s *t* test analyses for Figures 1, B, 2, D, 3, 9, and 10, K.

**Supplemental Data Set 2.** Tobacco primary and secondary metabolite profiling data.

**Supplemental Data Set 3.** Tobacco plastid tiling microarray design and ribosome profiling and transcriptome data.

**Supplemental Data Set 4.** One-way ANOVA reports generated using SigmaPlot14 software containing statistical analyses for Figure 10, B–I and Table 1.

## Supplementary Material

koac056_supplementary_data

## Notes

R.Z. designed the research with input from R.B. If not otherwise stated, Y.G. performed the experiments. Y.G. and H.M.Z. analyzed microarray data. M.A.S. and W.T. performed photosynthetic measurements. K.K. and O.S. performed quantitative PCR experiments. A.F., F.S., and F.A. measured metabolite levels. A.S. performed microscopy. R.Z. and Y.G. wrote the article with significant contribution from M.A.S and R.B. All authors approved the final version.

The author responsible for distribution of materials integral to the findings presented in this article in accordance with the policy described in the Instructions for Authors (https://academic.oup.com/plcell) is: Reimo Zoschke (zoschke@mpimp-golm.mpg.de).
