# Supplementary material for: Chloroplast translational regulation uncovers nonessential photosynthesis genes as key players in plant cold acclimation
Source: Plant Cell. 2022 Feb 16;34(5):2056–79. doi: 10.1093/plcell/koac056 (PMC9048916; doi:10.1093/plcell/koac056)
Supplement: koac056_supplementary_data [file koac056_supplementary_data.zip › TPC2021RA00599R2 Supplemental Figures and Tables Final_updated.pdf]

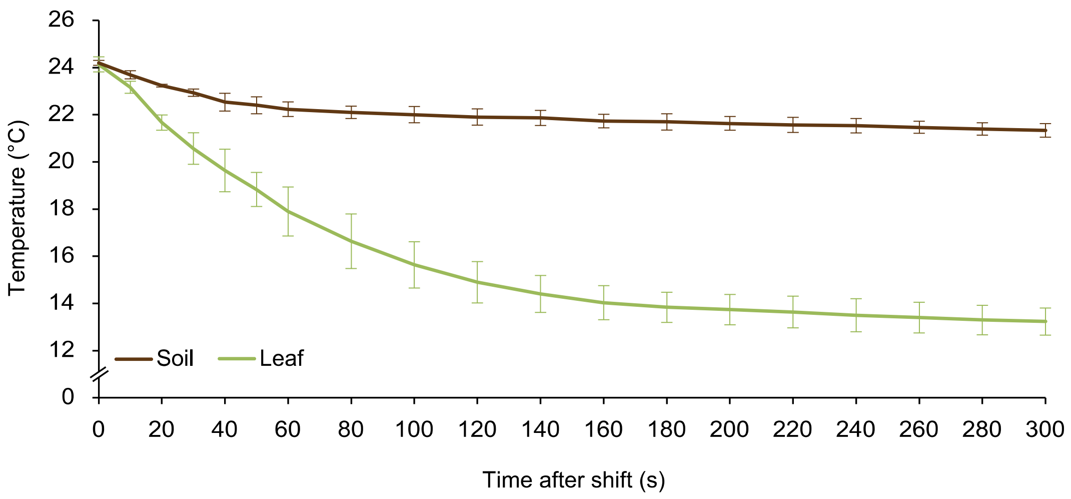

Supplemental Figure S1. Leaf and soil temperatures after cold shift.
Line plots represent the changes in tobacco leaf and soil surface temperature after cold shift. Error bars denote the standard deviation of three independently measured technical replicates (individual plants). (Supports Figure 1).

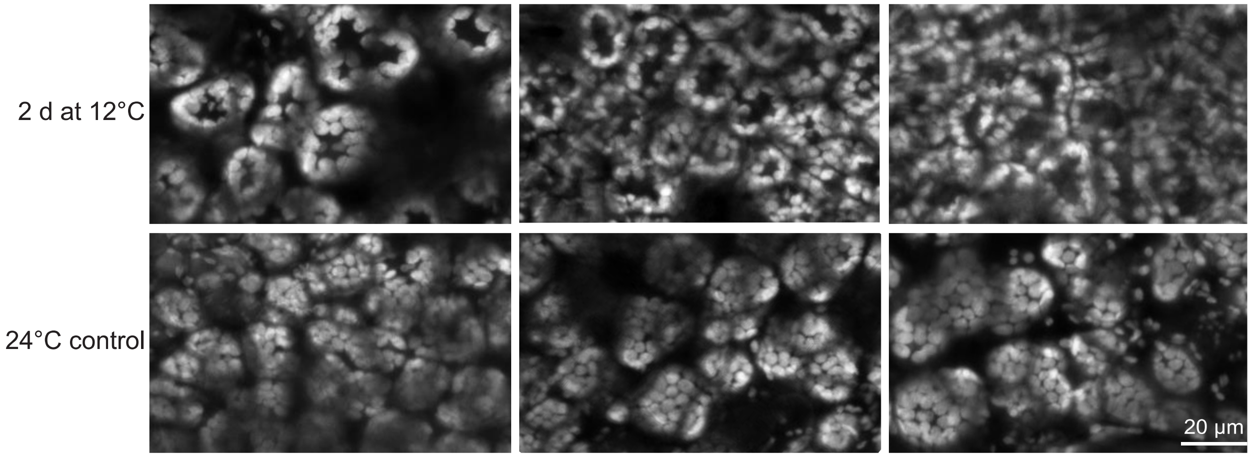

Supplemental Figure S2. Chloroplast movement after cold shift.
A top-down view of chloroplasts in mesophyll cells of three additional acclimating and control plants grown for 2 d after cold shift, as shown in Figure 1. Four acclimating and four control plants were analyzed (and one plant is shown in Figure 1).
(Supports Figure 1).

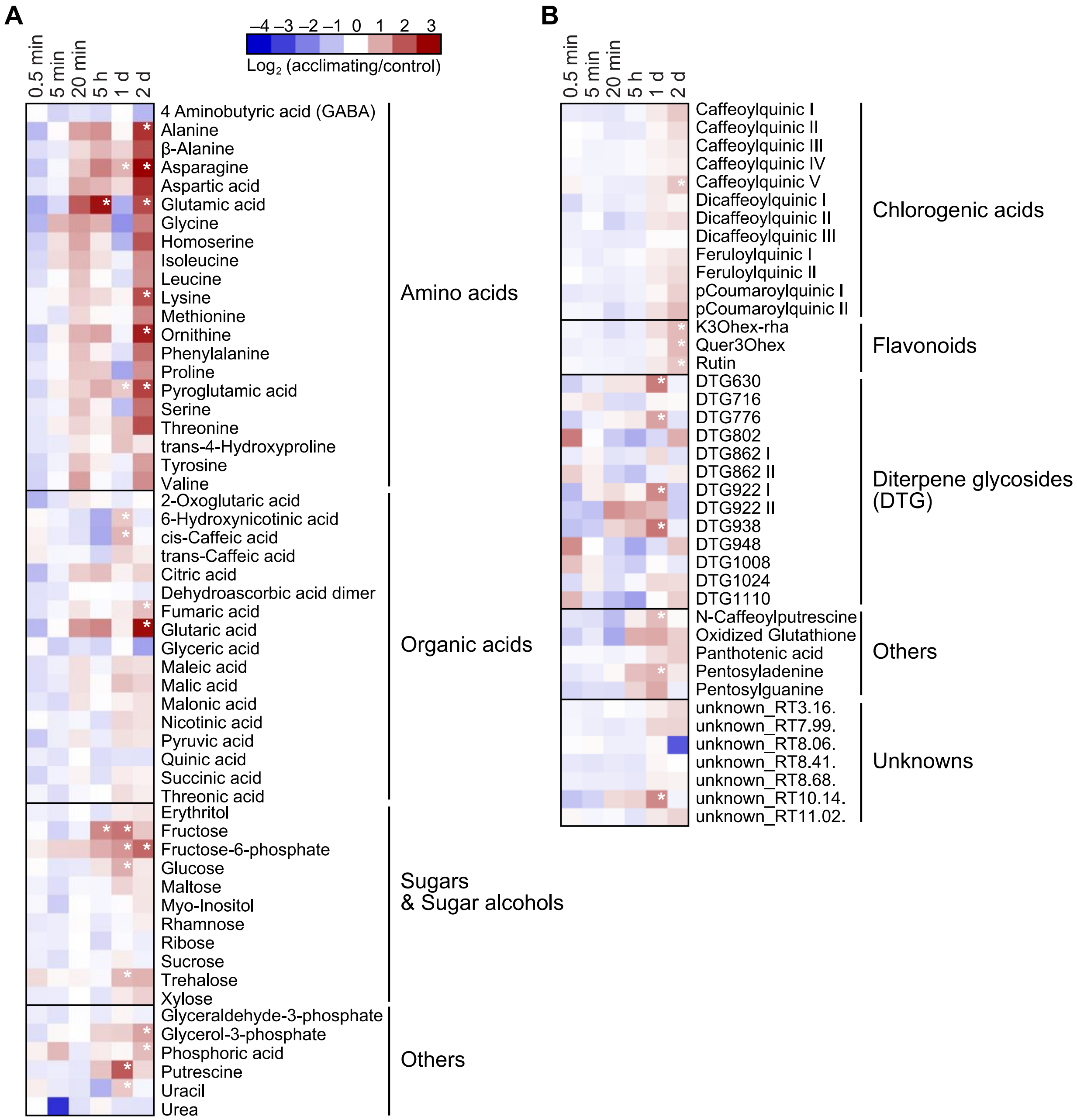

Supplemental Figure S3. Metabolite changes during cold acclimation.

(A-B) Heatmaps representing the Log_2-transformed fold-changes in the accumulation of primary metabolites (**A**) and secondary metabolites (**B**) at the indicated time points after cold shift. Results are shown for metabolites that were detected in at least three out of four independent biological replicates (i.e., individual plants, details in Methods, Supplemental Data Set S2). Relative upregulation and downregulation of accumulation is shown in red and blue, respectively, as indicated by the color scale. Metabolites are grouped according to the indicated categories. White asterisks mark greater than 1.5-fold fold change with statistical significance (q-value <0.1).
(Supports Figure 1).

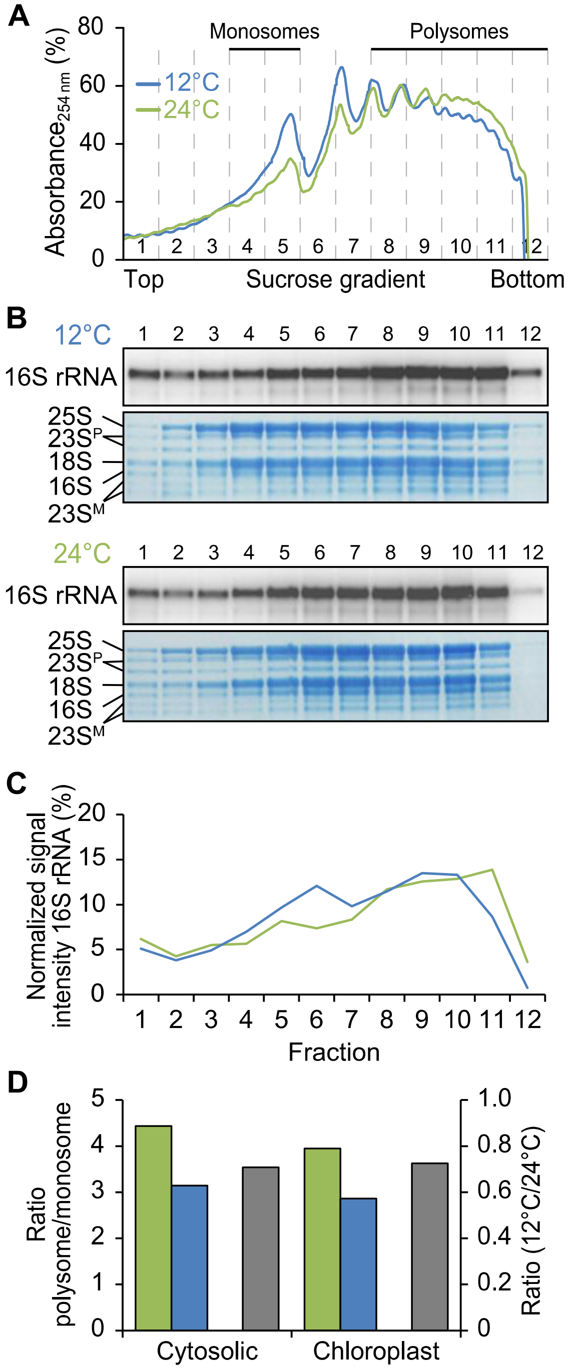

Supplemental Figure 4. Mild global reduction of cytosolic and chloroplast translation initiation after 20 min at low temperature.
(A) Polysome loading profiles of cytosolic ribosomes in acclimating and control plants (labeling details as in Figure 2; results were obtained from one sample; note that cytosolic ribosomes predominate the profiles, while chloroplast ribosomes represent a minor fraction). **(B)** RNA gel blot analyses demonstrating the distribution of chloroplast 16S rRNA in indicated gradient fractions from acclimating and control plants. **(C)** Quantification of 16S rRNA levels (as in Figure 2C). **(D)** Quantification of cytosolic and chloroplast translation activity (as in Figure 2D).
(Supports Figure 2).

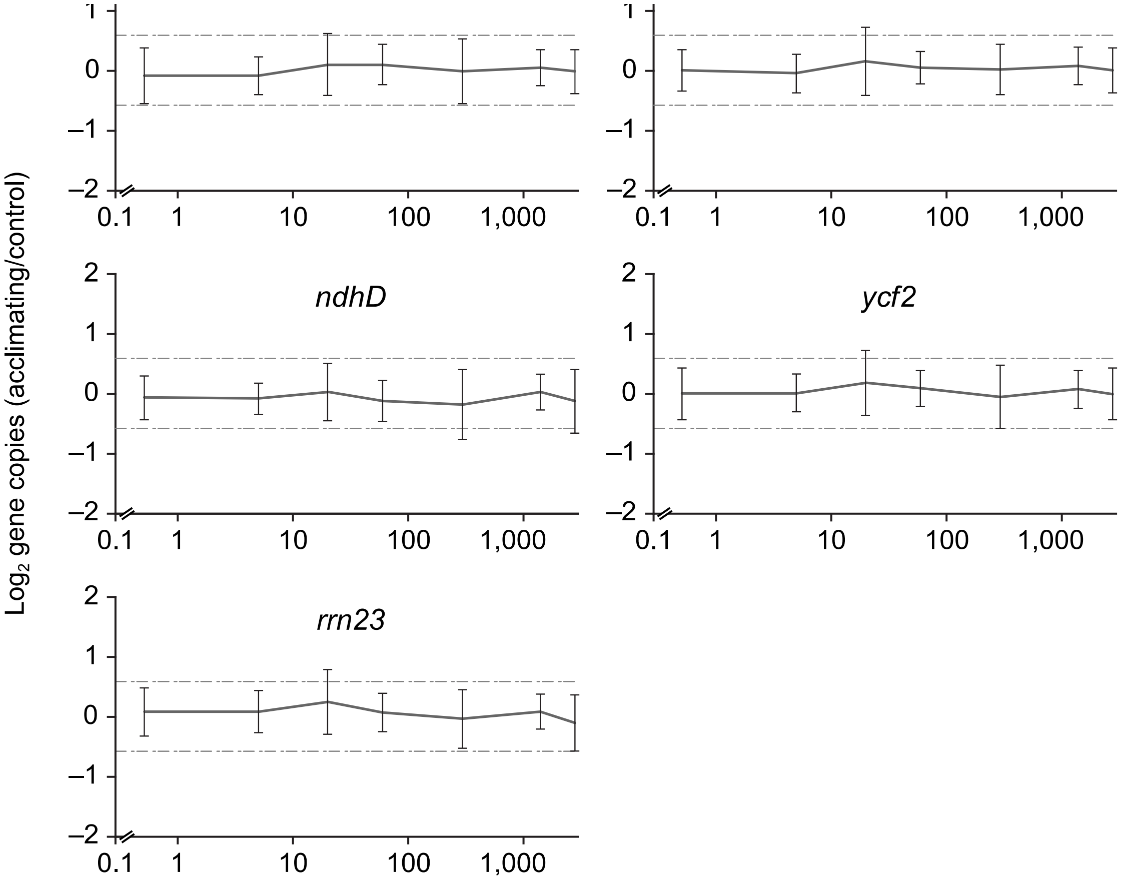

Supplemental Figure S5. Relative copy numbers of chloroplast genes during cold acclimation. Log_2-transformed fold-changes in chloroplast gene copy numbers, as measured by qPCR for the five indicated chloroplast genes at the designated time points after cold shift. Error bars denote the standard deviation of six independent biological replicates (i.e., individual plants). Note the logarithmic scale of the x-axis.
(Supports Figure 4).

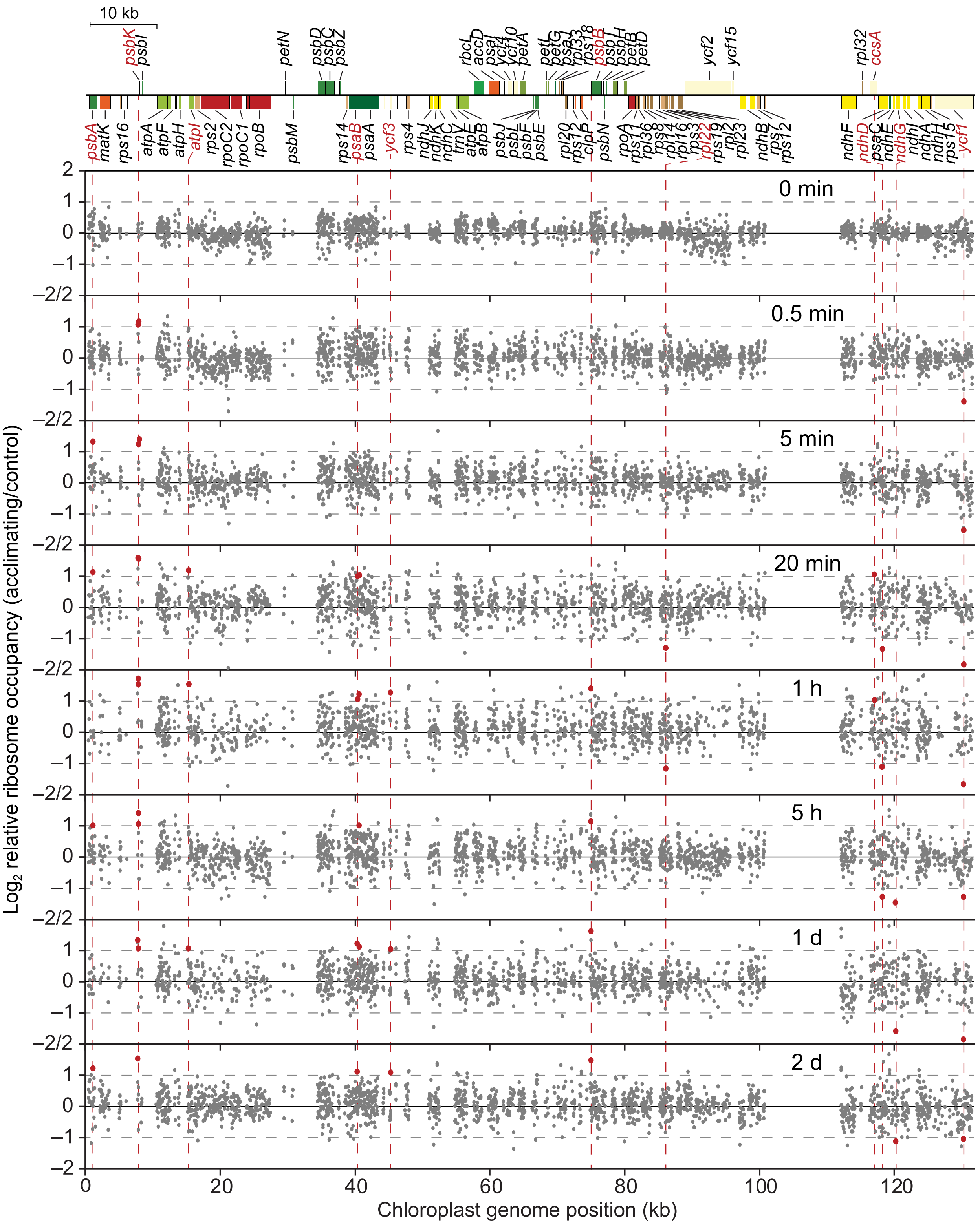

Supplemental Figure S6. Locally altered ribosome distribution in specific chloroplast reading frames during acclimation to low temperature.

Scatterplots representing the plastome-wide comparison of relative local ribosome occupancies between acclimating and control plants at the indicated time points after shift. The relative local ribosome occupancy

was calculated as the fraction of the ribosome footprint signal for each probe compared to the entire reading frame and plotted according to the position in the reading frame (see Methods for details). The results were obtained from three individual plants used as independent biological replicates, and the average values are plotted according to the position in the tobacco chloroplast genome. A physical map of the tobacco chloroplast genome was generated from the NCBI reference sequence Z00044.2 using OGDRAW (Greiner et al., 2019) and illustrates the protein-coding genes only. The map only shows one inverted repeat region of the chloroplast genome. Probes showing >two-fold changes between acclimating and control samples with statistical significance at two or more consecutive time points, as well as the genes they belong to, are plotted in bold and red color. The respective genome positions are marked with red dashed lines connecting to the corresponding genes in the map above. The significance of changes in relative local ribosome occupancy was assessed as described before (Schuster et al., 2020) using the empirical Bayes methods in the limma package (Smyth, 2004) and the *P*-values were adjusted to multiple testing according to the Benjamini-Hochberg procedure (Benjamini and Hochberg, 1995). Note that no statistically significant change was observed in relative local ribosome occupancies in the 0-min control samples.
(Supports Figure 8).

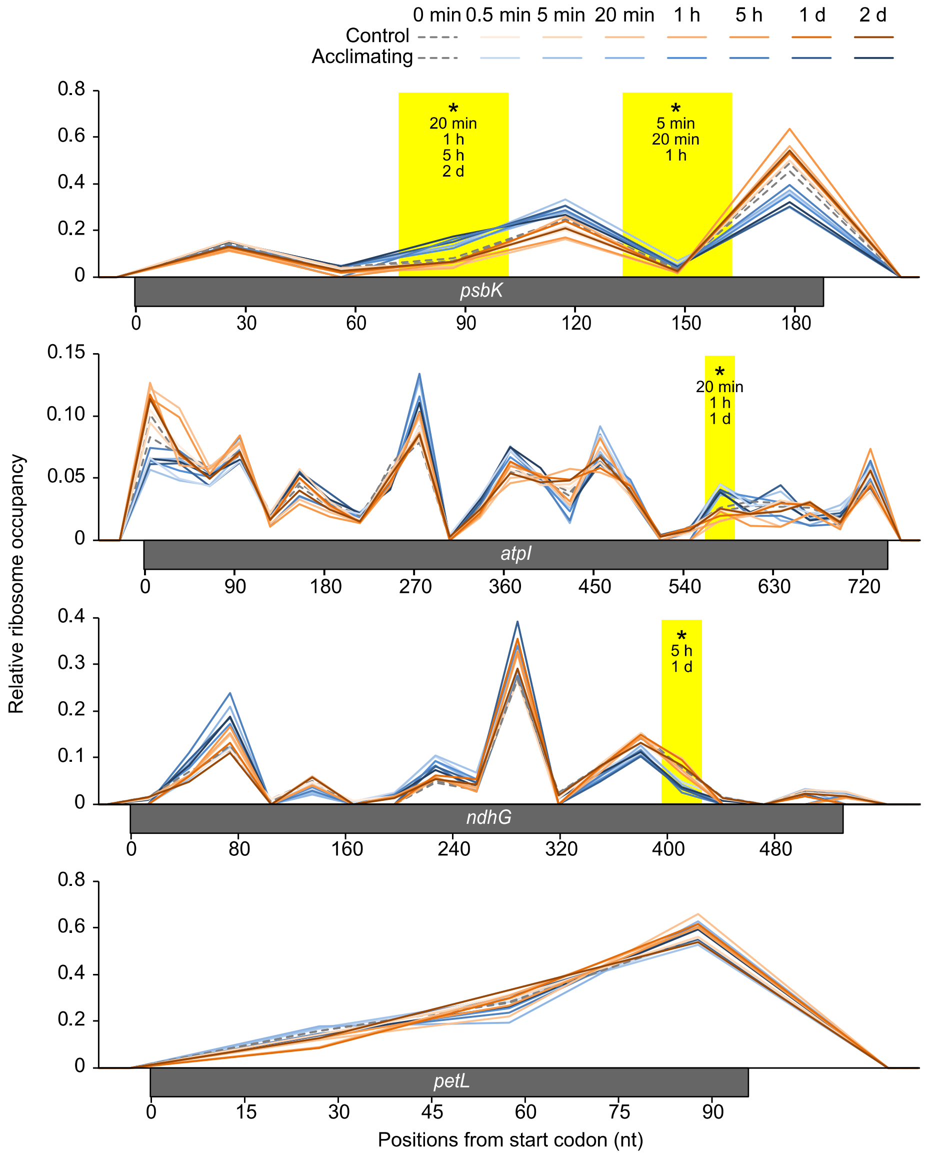

Supplemental Figure S7. Local ribosome redistribution in specific representative chloroplast reading frames during acclimation to low temperature.

Relative local ribosome occupancies in the indicated chloroplast reading frames for acclimating and control plants at the designated time points (legend on top of the plots). The positions of probes showing >two-fold changes between acclimating and control samples with statistical significance for two or more consecutive time points (indicated with asterisks) are shown as a 30-nt region shaded in yellow. Relative local ribosome occupancy on *petL* is shown as an example of unchanged local ribosome distribution after cold shift (while it has concomitantly increased overall ribosome occupancy, see Figure 5A-B). Dark gray boxes indicate protein-coding regions.
(Supports Figure 8).

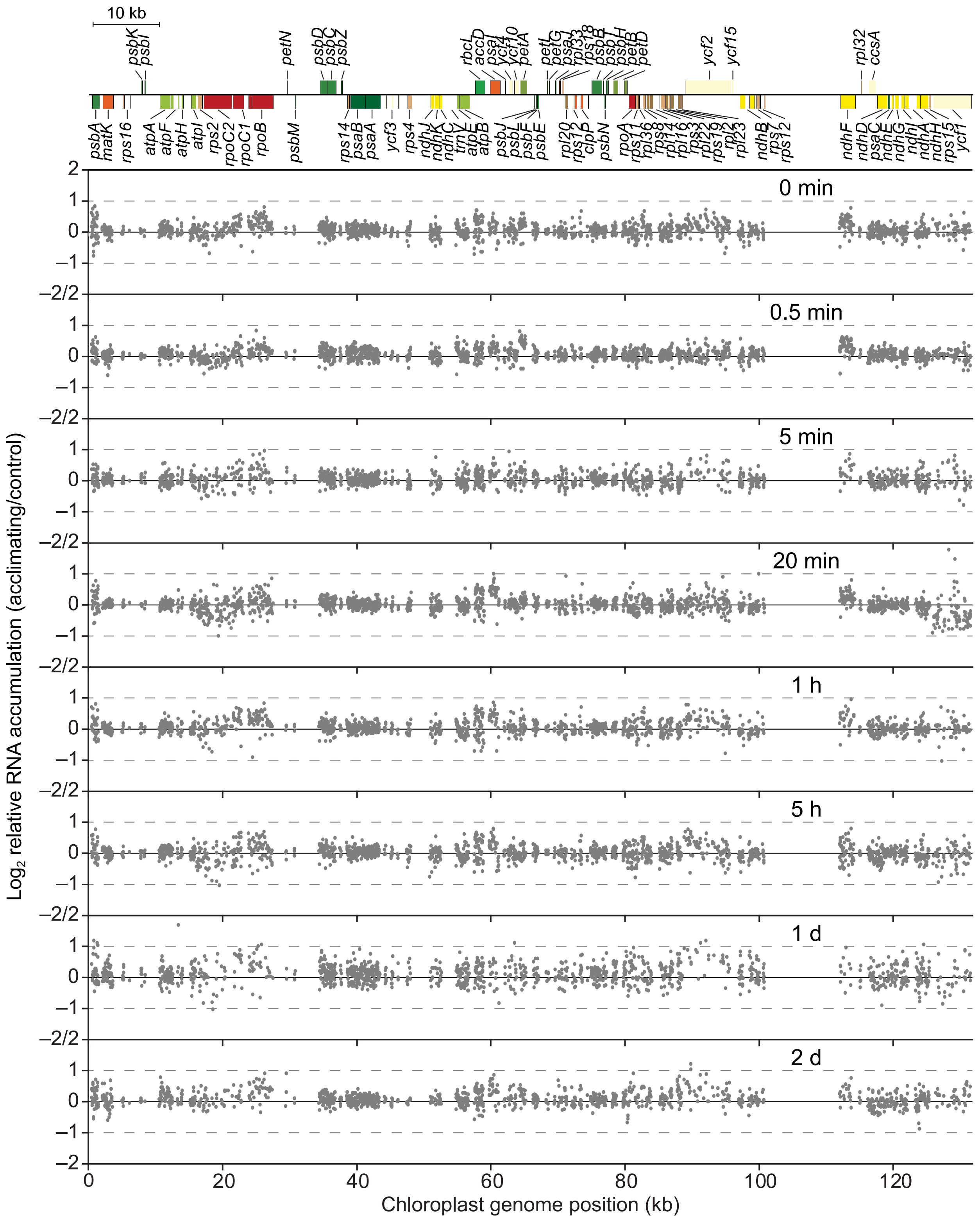

Supplemental Figure S8. Relative local chloroplast transcript coverage during acclimation to low temperature.

Scatterplots representing the plastome-wide comparison of relative local transcript coverage (fraction of microarray probe signal intensity per reading frame for transcriptome data; for details see Results and

Methods) between acclimating and control plants at the indicated time points after cold shift (labeling details as in Supplemental Figure S6). Note that no statistically significant change was observed.
(Supports Figure 8).

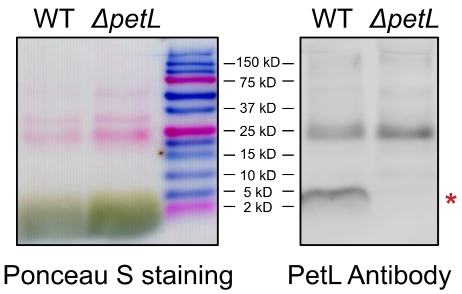

Supplemental Figure S9. Validation of the specificity of the anti-PetL antibody.
One µg of thylakoid proteins isolated from wild-type (WT) and *ΔpetL* plants were used for immunoblot analysis of PetL. Ponceau S staining of thylakoid proteins (left) and PetL signal (right, labeled with asterisk) are shown.
(Supports Figure 9).

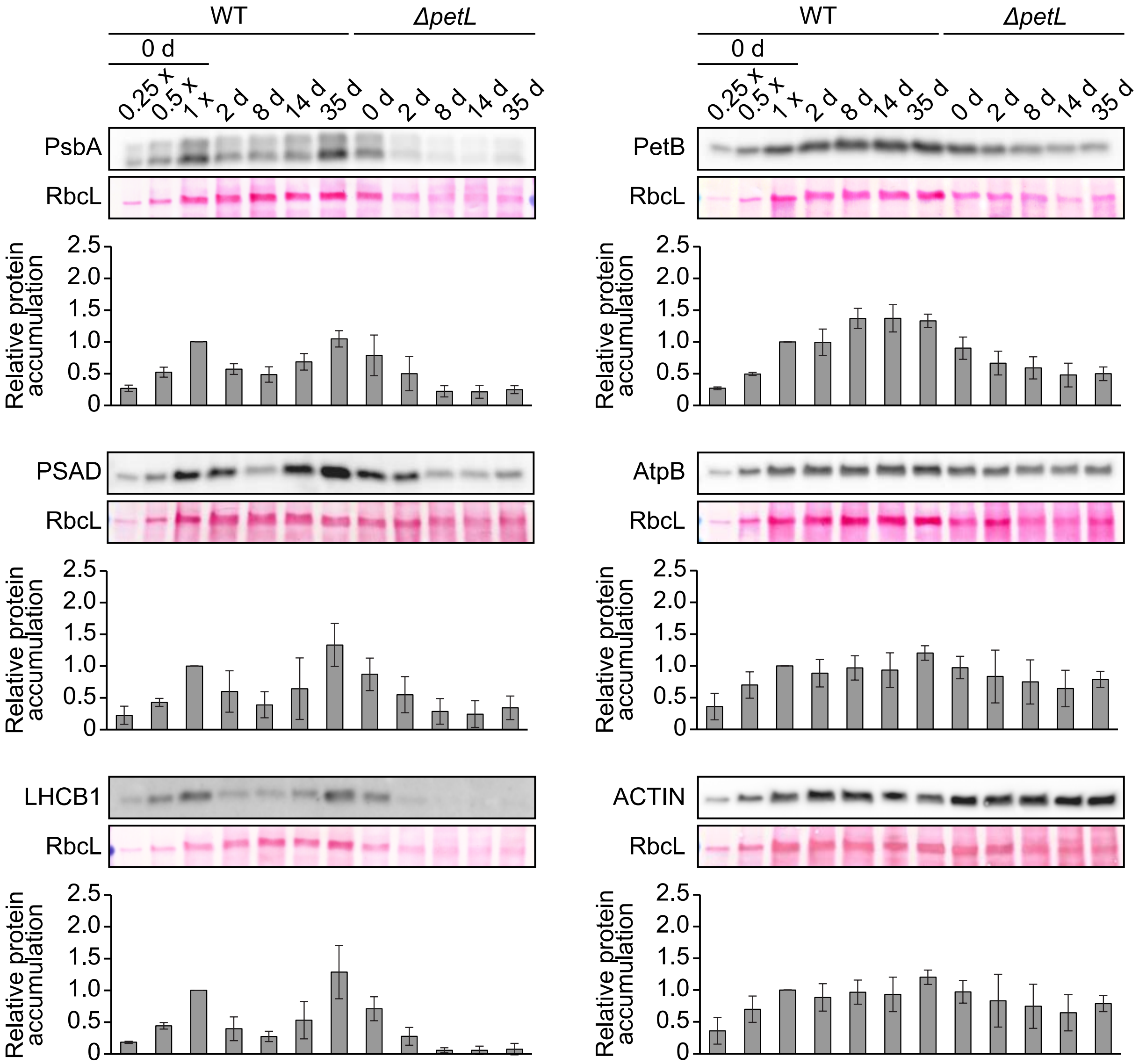

Supplemental Figure 10. Immunoblot analysis of core photosynthetic proteins in expanding leaves of cold-acclimating wild-type and *ΔpetL* plants.
Immunoblot analyses of the core subunits PsbA, PetB, PSAD, AtpB, of the photosynthetic complexes PSII, cyt b_6f, PSI, and ATP synthase, as well as the light harvesting complex protein LHCB1 and ACTIN as loading control in expanding leaves of cold-acclimating wild-type (WT) and *ΔpetL* plants at the indicated time points labeled on top. Four µg of total protein extracts were loaded for undiluted wild-type and mutant samples. In addition, a dilution series is shown for the wild-type sample at the 0-d time point on the left, as indicated above the blots. Ponceau S staining of RbcL is shown as loading control. For each of the probed proteins, one representative replicate out of three independent biological replicates (obtained from three individual plants) is shown. The band intensities were quantified using Image Lab software (Bio-Rad) and normalized to the intensity of the undiluted wild-type samples at the 0-d time point. Error bars denote the standard deviation of three independent biological replicates.
(Supports Figure 10).

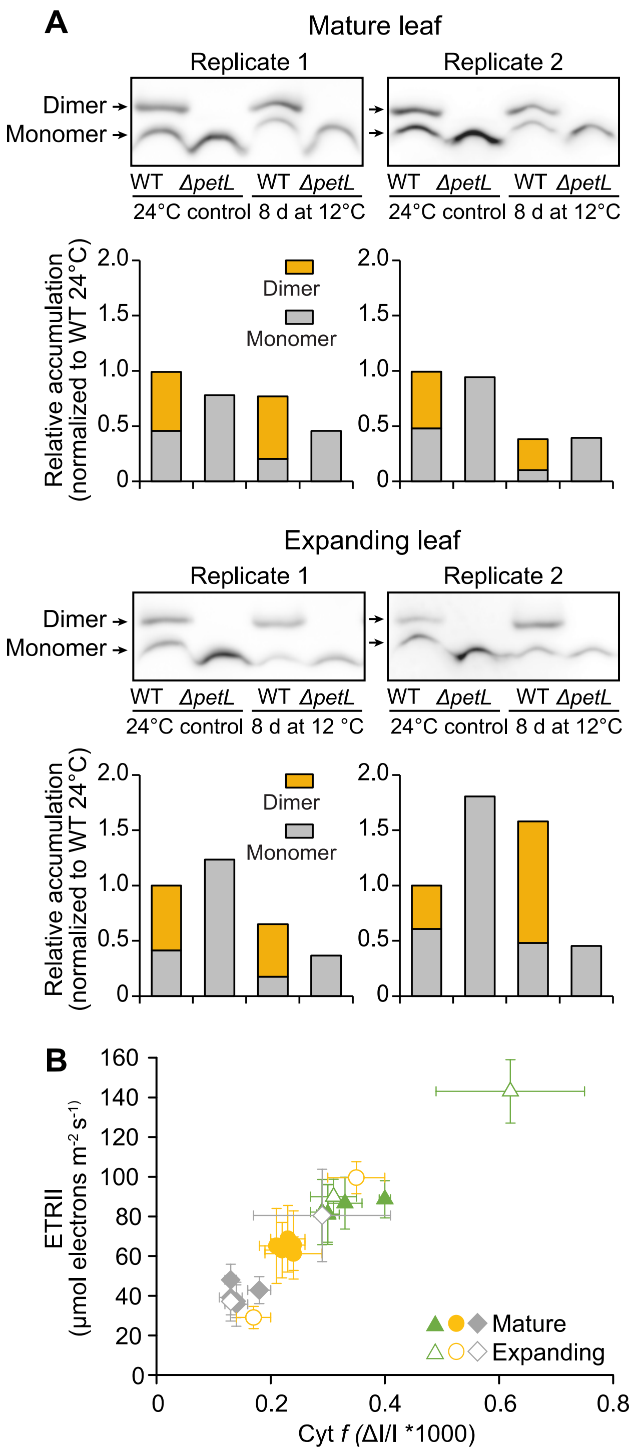

Supplemental Figure 11. Cyt *b₆f* dimer stability and activity in mature and expanding leaves of wild-type, *ΔpetL* and pRB8c plants after cold shift.

(A) Native polyacrylamide gel electrophoresis (PAGE) analysis of cyt b_6f complex accumulation in wild-type and *ΔpetL* plants. Native PAGE followed by immunoblots with PetB antiserum were performed with total protein extracts from mature (upper panels) and expanding (lower panels) leaves of wild-type (WT) and *ΔpetL* plants eight days after cold shift (12°C). Plants grown at 24°C of comparable developmental stage (similar size) were included as non-shifted controls. The observed monomeric and dimeric forms of the cyt b_6f complex are labeled. The band intensities of PetB in monomeric and dimeric cyt b_6f were quantified

using Image Lab software (Bio-Rad) and normalized to the summed intensity of both forms in the 24°C control wild-type sample. **(B)** Correlation of the maximum capacity of linear electron transport (ETRII) with in vivo contents of redox-active cyt *f* in mature and expanding leaves of wild-type, *ΔpetL* and pRB8c plants after cold shift (labeling details as in Figure 10; data obtained for each time point are shown in Supplemental Table S3). Data from expanding leaves of 8 d cold-exposed plants were excluded from the analysis for the following reason: the small size of these leaves caused pronounced developmental gradients in photosynthetic activity, as seen in the Imaging-PAM measurements averaging ETRII for the entire leaf area. cyt *f*, however, was only measured in one area in the middle of the leaf, therefore disabling direct data correlation. With increasing leaf age, these developmental gradients within each leaf increasingly vanished. (Supports Figure 10).

Supplemental Table S1. Mean and standard deviation parameters of probability density plots of local ribosome distribution and transcript coverage during cold acclimation.

Time point	Mean (Average distribution numbers)		Standard deviation (σ)	
	Ribosome	mRNA	Ribosome	mRNA
0 min	0.019	0.052	0.263	0.201
0.5 min	0.026	0.058	0.359	0.180
5 min	0.026	0.049	0.384	0.211
20 min	0.106	0.002	0.472	0.234
1 h	0.130	0.043	0.486	0.222
5 h	0.056	0.001	0.424	0.230
1 d	0.056	0.105	0.450	0.311
2 d	0.052	0.080	0.412	0.226

Supplemental Table S2. Primers used for qPCR.

Gene	Primer 1	Primer 2
ndhD	AGGCGGATTCTATCATAAGTCG	TCTCATGGATTTATTGGTGCTG
psbD	ACCAAAAATATTCTCTTAAACGAAGG	AAGGTTTTCATGAGGCTGATCT
rbcL	TTCCGGGTATTAGCAAAAGC	CCTACTACGGTACCAGAGTGAATATG
23S rDNA	TGTATTGCTCTCCCACAACC	ACCTGGCGAACTGAAACATC
ycf2	TGAATCGGAGTTTGAAGAAGG	AACTATGTGATTGAATAAATCCTCCTG
18S rDNA	GCCCTATCAACTTTCGATGGTA	CGAACCCTAATTCTCCGTCA

Supplemental Table S3. *in vivo* contents of redox-active cyt *f* and ETRII in wild-type, *ΔpetL* and pRB8c plants after cold shift.

Average values and standard deviation of *in vivo* content of redox-active cyt *f* and ETRII are shown for mature and expanding leaves of wild-type, *ΔpetL*, and pRB8c plants at indicated time points after cold shift. The results were obtained from the measurements of at least eight individual plants used as independent biological replicates.

**Mature leaves**	**cyt *f***			**ETRII**		
**Time point**	**WT**	***ΔpetL***	**pRB8c**	**WT**	***ΔpetL***	**pRB8c**
**1 d**	**0.30** ± 0.02	**0.23** ± 0.03	**0.18** ± 0.02	**81.0** ± 15.1	**68.7** ± 16.8	**42.8** ± 6.8
**2 d**	**0.29** ± 0.02	**0.24** ± 0.02	**0.14** ± 0.02	**82.2** ± 16.5	**65.6** ± 17.2	**35.7** ± 11.1
**4 d**	**0.30** ± 0.01	**0.21** ± 0.03	**0.13** ± 0.02	**81.5** ± 14.5	**65.1** ± 18.9	**39.1** ± 8.7
**8 d**	**0.33** ± 0.03	**0.22** ± 0.03	**0.13** ± 0.01	**86.7** ± 13.1	**63.0** ± 14.0	**48.0** ± 7.9
**14 d**	**0.40** ± 0.01	**0.24** ± 0.05	**0.14** ± 0.01	**88.7** ± 9.4	**61.3** ± 8.5	**37.2** ± 8.3
**Expanding leaves**	**cyt *f***			**ETRII**		
**Time point**	**WT**	***ΔpetL***	**pRB8c**	**WT**	***ΔpetL***	**pRB8c**
**14 d**	**0.31** ± 0.04	**0.17** ± 0.03	**0.13** ± 0.01	**90.0** ± 8.8	**29.0** ± 5.6	**37.1** ± 9.9
**35 d**	**0.62** ± 0.13	**0.35** ± 0.05	**0.29** ± 0.12	**143.1** ± 16.0	**99.6** ± 8.1	**80.5** ± 23.3

REFERENCES

Benjamini, Y. and Hochberg, Y. (1995). Controlling the false discovery rate: a practical and powerful approach to multiple testing. J R Stat Soc Series B Stat Methodol **57**: 289–300.

Greiner, S., Lehwark, P., and Bock, R. (2019). OrganellarGenomeDRAW (OGDRAW) version 1.3.1: expanded toolkit for the graphical visualization of organellar genomes. Nucleic Acids Res **47**: W59–W64.

Schuster, M., Gao, Y., Schöttler, M.A., Bock, R., and Zoschke, R. (2020). Limited responsiveness of chloroplast gene expression during acclimation to high light in tobacco. Plant Physiol. **182**: 424–435.

Smyth, G.K. (2004). Linear models and empirical bayes methods for assessing differential expression in microarray experiments. Stat. Appl. Genet. Mol. Biol. **3**: Article3.
